# Cross-Over Application of Algerian Dairy Lactic Acid Bacteria for the Design of Plant-Based Products: Characterization of *Weissella cibaria* and *Lactiplantibacillus plantarum* for the Formulation of Quinoa-Based Beverage

**DOI:** 10.3390/microorganisms12102042

**Published:** 2024-10-09

**Authors:** Radjaa Cirat, Zineb Benmechernene, Hülya Cunedioğlu, Mariacinzia Rutigliano, Angela Scauro, Khaled Abderrahmani, Kihal Mebrouk, Vittorio Capozzi, Giuseppe Spano, Barbara la Gatta, Maria Teresa Rocchetti, Daniela Fiocco, Mariagiovanna Fragasso

**Affiliations:** 1 Laboratory of Applied Microbiology, Department of Biology, Faculty of Natural Sciences and Life, University of Oran, 1 Ahmed Ben Bella, Oran 31100, Algeria; cirat.radjaa@edu.univ-oran1.dz (R.C.); benmechernene.zineb@univ-oran1.dz (Z.B.); kihalm@gmail.com (K.M.); 2 Department of Agricultural Sciences, Food, Natural Resources and Engineering (DAFNE), University of Foggia, 71122 Foggia, Italy; hulya.cunedioglu@unifg.it (H.C.); mariacinzia.rutigliano@unifg.it (M.R.); angela.scauro@unifg.it (A.S.); giuseppe.spano@unifg.it (G.S.); barbara.lagatta@unifg.it (B.l.G.); mariagiovanna.fragasso@unifg.it (M.F.); 3 Scienzanova S.r.l., Via Enrico Mattei 85-87, 86039 Termoli, Italy; 4 Centre National de Recherche et de Développement de la Pêche et D’aquaculture (CNRDPA), 11, Boulevard Colonel Amirouche, Tipaza 42415, Algeria; khaled_abderrahmani@yahoo.fr; 5 Institute of Sciences of Food Production, National Research Council of Italy (CNR), C/o CS-DAT, Via Michele Protano, 71121 Foggia, Italy; 6 Department of Clinical and Experimental Medicine, University of Foggia, 71122 Foggia, Italy; mariateresa.rocchetti@unifg.it (M.T.R.); daniela.fiocco@unifg.it (D.F.)

**Keywords:** lactic acid bacteria, non-cow milk, probiotic, antimicrobial activity, camel milk, fermentation, sustainability, lactobacilli, quality, safety

## Abstract

The food industry constantly seeks new starter cultures with superior characteristics to enhance the sensory and overall quality of final products. Starting from a collection of Algerian dairy (goat and camel) lactic acid bacteria, this work focused on the exploration of the technological and probiotic potential of *Weissella cibaria* (VR81 and LVT1) and *Lactiplantibacillus plantarum* R12 strains isolated from raw camel milk and fermented milk, respectively. These bioactive strains were selected for their high performance among ten other LAB strains and were used as starter cultures to develop a novel and nutritionally enhanced dairy-like plant-based yogurt using quinoa (*Chenopodium quinoa* Willd) as a raw matrix. The strains were evaluated for their antagonistic effects against *Listeria innocua*, *Listeria ivanovii*, *Staphylococcus aureus*, *Escherichia coli*, *Salmonella enterica*, and *Pseudomonas aeruginosa*, resilience to acidic and osmotic challenges, and tolerance to gastrointestinal mimicking conditions (i.e., pepsin and bile salt). Their aggregation and adhesion profiles were also analyzed. Furthermore, *L. plantarum* and *W. cibaria* were tested in single and co-culture for the fermentation and biocontrol of quinoa. The strains exhibited probiotic properties, including a high potential for biocontrol applications, specifically against *L. innocua* and *P. aeruginosa* (20 mm diameter zone with the neutralized cell-free supernatant), which disappeared after protease treatment, suggesting that bioactive peptides might be responsible for the observed antimicrobial effect. Additionally, they demonstrated resilience to acidic (pH 2) and osmotic challenges (1M sucrose), tolerance to gastro-intestinal conditions, as well as good aggregation and adhesion profile. Furthermore, the strains were able to produce metabolites of interest, such as exopolysaccharide (yielding up to 4.7 mg/mL) and riboflavin, reaching considerable production levels of 2.5 mg/L upon roseoflavin selection. The application of *W. cibaria* and *L. plantarum* as primary starters (both in single and co-culture) for fermenting quinoa resulted in effective acidification of the matrix (ΔpH of 2.03 units) and high-quality beverage production. in vivo challenge tests against *L. innocua* showed the complete inhibition of this pathogen when *L. plantarum* was included in the starter, either alone or in combination with *W. cibaria*. Both species also inhibited *Staphylococcus* and filamentous fungi. Moreover, the co-culture of mutant strains of *L. plantarum* R12d and *W. cibaria* VR81d produced riboflavin levels of 175.41 µg/100 g in fermented quinoa, underscoring their potential as starters for the fermentation, biopreservation, and biofortification of quinoa while also displaying promising probiotic characteristics.

## 1. Introduction

LAB are Gram-positive, non-spore-forming, aerotolerant microorganisms that can ferment carbohydrates to produce lactic acid as the main final product. Such a large bacterial group comprises species largely used in the fermentation of food, where they contribute to enhancing the flavor, texture, nutritional value and shelf life of the final product [1]. Commercial LAB starter cultures exhibit diverse and desirable metabolic properties, including acidification and proteolytic activity, antagonism against deleterious/detrimental microbes, and the capability to produce bioactive compounds, including exopolysaccharides and vitamins. Many LAB strains are considered to be probiotic, i.e., “live microorganisms that, when administered in sufficient quantities, provide health benefits to the host” [2]. Probiotics can be incorporated into foods as supplements, hence offering consumers health advantages such as maintaining intestinal microbiota eubiosis, reducing cholesterol levels, strengthening immune defenses, and treating or preventing gastrointestinal disorders [3]. Among others, a key attribute of LAB is their ability to biosynthesize water-soluble B-group vitamins (i.e., folic acid, riboflavin, niacin, and cobalamin), essential nutrients that the human body cannot produce [4]. Riboflavin, also known as vitamin B2, is a water-soluble vitamin naturally synthesized by plants. A deficiency in riboflavin can lead to various health issues, including cardiac and skin disorders, migraines, and disruptions in sugar metabolism [5]. Interestingly, riboflavin-overproducing LAB can be used as starter cultures for in situ biofortification during food fermentations, providing high levels of riboflavin that can meet the daily intake requirements ranging from 0.3 to 1.5 mg/day [6]. Considering the importance of dairy matrices for isolating indigenous LAB, camel and goat milk serve as promising sources of strains with desirable biofunctional properties [7,8]. In 2020, Algeria’s camels produced 15,080 tons of milk, emphasizing their economic importance in the livestock sector [9]. Similarly, the significance of goat milk is increasingly recognized worldwide, with products like yogurt, butter, cheese, and ice cream gaining popularity due to their high nutritional value [10,11]. Goat milk, particularly in Algeria and Sub-Saharan Africa, offers advantages over cow’s milk, such as higher vitamin content and superior cheese yield [12]. Both camel and goat milk are important reservoirs of lactic acid bacteria (LAB). The dominant lactic acid bacteria (LAB) species found in camel and goat milk include *Limosilactobacillus fermentum*, *Lactiplantibacillus plantarum*, *Lacticaseibacillus casei*, *Lactococcus lactis* subsp. *lactis*, *Enterococcus faecium*, and *Streptococcus thermophilus* in camel milk [13], while goat milk is primarily characterized by *Lactococcus* spp., lactobacilli, *Leuconostoc*, and *Enterococcus* [14]. LAB from these milks exhibit probiotic properties, including antioxidant and anti-inflammatory effects, making them valuable for food fermentation and biofortification [15,16]. The food and feed industries extensively employ lactic acid bacteria (LAB) for their capacity to ferment, enhance sensory attributes, and improve the overall quality and safety of the final product. Recent research has explored the significant role of lactic acid bacteria (LAB) in enhancing the active substances and functional properties of plant-based products. The fermentation process performed by lactic acid bacteria (LAB) constitutes a novel strategy for developing plant-based foods, enhancing their nutritional value and functional properties [17].

Fermented foods and plant-based beverages are products that offer significant nutritional and functional benefits while positively impacting human health [18]. In this context, plant-based diets could offer significant benefits, reducing risks associated with animal-based products and increasing the intake of dietary fiber—comprising non-digestible and non-absorbable carbohydrate polymers plays a crucial role in enhancing gut microbiota diversity and promoting the synthesis of beneficial metabolites [19]. However, despite the growing interest in plant-based alternatives, achieving desirable attributes remains a significant challenge in their development. An extensive understanding of the fermentation process in plant-based dairy substitutes is crucial for developing appealing products for consumers transitioning to new alternatives [20]. This has boosted interest in technological features such as EPS-producing LAB that are essential in the fermentation process of plant-based foods such as yogurt-like beverages and cheese alternatives. The polysaccharides they produce enhance both the texture and sensory qualities of these plant-derived products while also extending their shelf life [21]. Therefore, the investigation into in situ EPS production is highly appealing for producers of plant-based foods (e.g., cereal) aiming to mimic the characteristics of fermented dairy products. This involves employing both pure and mixed LAB cultures to enhance the texture of non-dairy food products through EPS formation [22]. The lactic fermentation of cereals like maize, millet, barley, oats, rye, wheat, rice, and sorghum into beverages is a long-standing tradition in Africa. Globally, there is ongoing exploration into developing new lactic-fermented products, recognized as an effective way to enhance the daily intake of fresh vegetables and fruits [21]. Quinoa (*Chenopodium quinoa* Willd.) is a tetraploid herbaceous plant belonging to the Dicotyledoneae class. Recognized for their rich nutritional profile, quinoa seeds are gluten-free and provide essential amino acids (lysine, tryptophan, and cysteine), vitamins (E, B, C), and minerals such as calcium, iron, manganese, magnesium, copper, and potassium. Furthermore, quinoa is regarded as one of the best vegetal protein sources, as its values are close to those specified by the Food and Agriculture Organization (FAO) [23]. Additionally, quinoa, known for its high-fiber content and antioxidants, including polyphenols, is suitable for individuals with celiac disease or gluten-related disorders. The recent literature has documented some biotechnological methods for developing quinoa-based fermented products through lactic acid bacteria fermentation [24,25,26]. Despite the potential benefits of quinoa-based fermented products, several challenges remain to be addressed to ensure broader consumer acceptance and improve their overall quality, selecting starter cultures capable of improving global quality and safety. During industrial processing, the vitamin content of these products tends to decrease, thereby diminishing their overall nutritional value [27]. While quinoa’s high nutritional profile makes it an ideal ingredient for such applications, the sensory attributes remain a major obstacle in developing products with comparable sensory appeal to dairy-based products [28]. Microbiological safety is yet another concern, as these products sometimes exhibit high microbial loads, thereby increasing the risk of pathogen exposure [28]. The presence of bacterial contamination is difficult to control with mild heat treatments, as the cells often exhibit increased heat tolerance at lower water activities [29]. A specific safety issue in quinoa fermentation is the spontaneous growth of *Enterococcus*, which can originate from quinoa and contribute to health risks for consumers [30]. Therefore, addressing these challenges is crucial for optimizing the safety, nutritional quality, and consumer acceptance of quinoa-based fermented products. Cross-over fermentations involve introducing a microorganism from a traditional fermentation process to a new substrate or combining it with a new microbial partner in a mixed culture [31]. 

This study addresses the growing interest in the crossover application of dairy lactic acid bacteria for the fermentation of a plant-based matrix. In fact, there are limited literature reports on dairy–plant crossovers, particularly in the context of quinoa-based fermented beverages. Moreover, while some studies focus on the chemical, rheological, and nutritional properties of quinoa yogurt-like beverages [32], no previous research has investigated the antimicrobial potential of dairy lactic acid bacteria within this emerging sector. Therefore, our study explores the in vivo antagonistic abilities of selected LAB strains against spoilage and pathogenic microorganisms to enhance the biocontrol in quinoa beverages by exploiting the fermentation process. These strains were also selected to offer probiotic benefits. Probiotic characterization involved extensive testing, including antimicrobial potential, thermal stability of antimicrobial substances, resistance to low pH, bile salts, and pepsin, and evaluating adhesion capacities, auto-aggregation, co-aggregation, and safety features like hemolysis and antibiotic susceptibility. The technological characterization focused on enzymatic activities and the ability of the strains to biosynthesize valuable substances such as organic acids, exopolysaccharides, and vitamin B2. In the final phase, we evaluated the potential of *Lactiplantibacillus plantarum* and *Weissella cibaria* as starter cultures, testing them in both single and co-culture for fermenting quinoa flour, leading to the development of a riboflavin bio-fortified quinoa beverage.

## 2. Materials and Methods

### 2.1. LAB’s Strains Isolation and Preliminary Characterization

#### 2.1.1. Isolation of LAB from Algerian Dairy Products 

LAB strains producing antimicrobial substances were isolated from different Algerian dairy products, i.e., raw camel and goat milk, *jben* (Traditional goat cheese) and *leben*, made of fermented camel’s and goat’s milk, respectively. Bacterial cultures were obtained by streaking ten-fold dilutions of each dairy sample on three distinct culture media. De Man–Rogosa–Sharpe (MRS, Biolife Italiana S.R.L, Milan, Italy) agar (pH 5.4, supplemented with 0.05% *w*/*v* cysteine) enabled the selective isolation of lactobacilli, Mayeux, Sandine & Elliker (MSE, Biolife Italiana S.R.L, Milan, Italy) agar facilitated the selection of *Leuconostoc* and *Weisella*, while M17 (Oxoid, London, UK) agar supported *Lactococcus* growth. The incubation conditions varied (30 °C aerobically for *Leuconostoc* and *Weissella*, 37 °C anaerobically for lactobacilli). Gram-positive and catalase-negative isolates were chosen after initial phenotypic analysis, stored at −80 °C in 30% (*w*/*v*) Glycerol–MRS broth, and subjected to two rounds of spreading on MRS agar before experiments. 

Based on the results from the preliminary inhibitory assay using agar disk assay against *Escherichia coli*, *Staphylococcus aureus*, *Salmonella enterica*, *Pseudomonas aeruginosa*, and *Listeria innocua*, twelve bioactive strains were selected and subjected to phenotypic and morphological characterization.

#### 2.1.2. Physiological and Biochemical Characterization for a Preliminary Selection of LAB Strains of Interest

The physiological characteristics of twelve bacteriocin-like-producing LAB strains were determined using a series of tests: growth at different temperatures (4 °C, 15 °C, 37 °C, 45 °C), growth at different pH levels (4.8 to 6.8), tolerance to 3% and 6.5% of NaCl, thermo-resistance at 55 °C and 63.5 °C for 15 min, CO_2_ production from glucose, arginine hydrolysis, on M16BCP medium (Oxoid, London, UK). The fermentation of carbohydrates was studied by using API 50 CH strips (bioMérieux, Marcy l’Etoile, France) following the manufacturer’s recommendations.

#### 2.1.3. Genetic Identification of Selected Strains of Interest

The genomic DNA from twelve LAB strains was isolated and purified using the Omega Biotek E.Z.N.A. DNA extraction kit (Norcross, GA, USA), according to the manufacturer’s protocol. The genomic DNA was PCR amplified using the universal primer pair p8FPL (forward: 5′-AGTTTGATCCTGGCTCAG-3′) and p806R (reverse: 5′-GGACTACCAGGGTATCTAAT-3′), for sequencing of the 16S rDNA region, leading to the identification of the LAB species (PP952045-PP452046-PP952008-PP952004-PP952001-PP951999-PP951997-PP951993-PP93976-PP915623-PP915563-PP915466). Amplification conditions included initial denaturation at 94 °C for 7 min, followed by 35 cycles of denaturation (94 °C for 60 s), annealing (55 °C for 60 s), and extension (72 °C for 60 s), with a final extension at 72 °C for 15 min.

### 2.2. Probiotic Evaluation of the Bioactive Strains

The technological and probiotic properties of the most performant LAB strains were also examined to evaluate their aptitude for being applied as an indigenous starter culture.

#### 2.2.1. Inhibition Spectra

The selected LAB strains were further screened for their aptitude to produce antimicrobial substances via the agar well diffusion method, as described by Tagg et al. (1979) [33,34]. The CFS of the 12 LAB strains was tested against the following indicator microorganisms: *L. innocua* ATCC 33090, *L. ivanovii* ATCC 19119, *P. aeruginosa* ATCC 27853, *S. aureus* ATCC 25923, *E. coli* ATCC 25922, and *S. enterica* ATCC 1428. Target microorganisms were cultured on BHIB (Brain Heart Infusion Broth, Biolife Italiana S.R.L, Milan, Italy) and incubated at 37 °C for 24 h. Briefly, an aliquot of 60 µL of the CFS from each LAB strain, obtained after centrifugation at 8000 rpm for 10 min, was filled into 6 mm diameter wells formed in BHI agar, previously inoculated (1%, *v*/*v*) with indicator strains at an optical density of 0.1 (OD600). Inhibition zones were measured following 24 h incubation at 37 °C. *Leuconostoc mesenteroides* B7 [35] and *L. plantarum 299* V [36] were used as controls for the antagonistic assay and the adhesion to human enterocyte-like cells, respectively. The strains were both cultivated in MRS broth and incubated at 37 °C.

#### 2.2.2. Detection of Proteinaceous Nature of the Inhibitory Agent

To determine the proteinaceous nature of the inhibitory substance, the proteinase sensitivity of LAB strains with the broadest antimicrobial spectrum was evaluated. The cell-free supernatants (CFSs) obtained after centrifugation at 8000 rpm for 10 min of each LAB isolate were neutralized to pH 6 with 1N NaOH and then filtered through a 0.45 Millipore filter (0.45 µm, Sigma-Aldrich, Steinheim, Germany). Different proteolytic enzyme solutions were prepared with the appropriate buffer (i.e., pepsin with 0.02 M HCL, pH 2; trypsin with 0.1 M phosphate buffer, pH 6; Proteinase K with 10 Mm Tris-HCL, pH 7.5, Sigma-Aldrich, Steinheim, Germany). Aliquots of each neutralized cell-free supernatant (NCFS) were treated with different enzymes at a final 1 mg/mL concentration. Incubation was carried out at 37 °C for one hour, followed by enzyme inactivation at 65 °C for 10 min. The antimicrobial effect of the protease-treated NCFS and non-treated NCFS (without enzyme) was tested using the agar well diffusion bioassay, as described above [35]. To exclude the inhibitory effect of hydrogen peroxide, indicator microorganisms (*Listeria innocua*, *Listeria ivanovii*) with catalase enzyme were used and inoculated in depth to minimize oxygen impact. 

#### 2.2.3. Assessment of Heat Treatment, pH, and Chemical Detergents on the Bacteriocin Activity

To evaluate the thermal stability of the inhibitory agent at high temperatures, the CFS of the selected isolates was heated at different temperatures, 60, 70, 80, and 90 °C for 30 min and at 100, as well as 120 °C for 15 min. The antimicrobial activity of the heated CFS was then evaluated via the agar well diffusion bioassay with a nonheated CFS (no heat treatment) as a negative control. Furthermore, the incidence of pH on the activity of the bacteriocin-producing strains was tested by adjusting the pH levels of each CFS ranging from 3 to 9 (adjusted with 1 N HCL or 4 N NaOH) and incubating for two hours at 37 °C; the sensitivity of the antimicrobial compound to detergents was investigated by treating NCFS with 1% of urea and tween 80 and then incubating for two hours at 37 °C. The stability of the antimicrobial activity was verified after each test via the agar well diffusion assay, as described above [37]. 

#### 2.2.4. Acidifying Activity, Milk Coagulation and pH Tolerance

Acidification capacity was studied using pure cultures of the selected LAB strains. In brief, 1% of the overnight culture of each LAB strain was inoculated in 10 mL of skim milk and plant-based beverages, including coconut milk, oat milk, soy milk, and quinoa beverages. The incubation was performed at 37 °C for the *L. plantarum* strains and at 30 °C under aerobic conditions for *W. cibaria* strains for 18 h. The precultures were then inoculated separately in 100 mL of each beverage supplemented with 0.3% yeast extract. The total volume was divided into ten tubes of 10 mL. The pH evolution was measured with a pH meter at the beginning of inoculation and every 2 h of incubation for 24 h. One negative control (non-inoculated UHT skim milk) was also studied.

Milk coagulation was investigated by inoculating 2% from an 18 h culture of each LAB strain in 10 mL of skim milk and incubating the culture solutions at 30 °C for five days. The cultures were visually checked after 6, 12, 24 h, 2, 3, 4, and 5 days of incubation in order to determine the necessary time for the appearance of the coagulum [38].

The bacteriocin-producing LAB strains were also tested for their ability to grow at low pH levels using a slightly modified method presented by Kiani et al. [39]. After centrifugation, the pellets of LAB strains (10^8^ CFU/mL) were resuspended in an MRS broth and adjusted to pH 2, pH 3, and pH 4, respectively. The suspensions were then incubated at 37 °C for 3 h. Viable bacterial cell counts were enumerated on MRS agar at both 0 and 3 h of incubation.

#### 2.2.5. Bile Salt Tolerance and Bile Salt Hydrolysis 

The resistance to bile salts was investigated according to the previous method of Kiani et al. [39]. In this approach, an MRS broth containing varying concentrations of oxgall (0.5%, 1%, 2%, *v*/*v*) was inoculated with a LAB pellet and incubated for 4 h at 37 °C. Subsequently, viable cell counts were determined on MRS agar at both 0 and 4 h of incubation. The acid and bile tolerance results were calculated and represented as the survival rate using the following equation:

Survival Rate (%): [number of colonies after treatment)/initial number of colonies (Before treatment)] × 100%.

Finally, bile salts hydrolysis was also tested by streaking 100 uL of LAB suspension (18 h culture 10^8^ UFC/mL) on MRS agar containing 0.5% Oxgall and incubating at 30 °C for 24 to 48 h. Hydrolysis is defined by the morphological alteration of LAB colonies surrounded by a precipitation zone compared with negative control (MRS without Oxgall) [35].

#### 2.2.6. Antibiotic Susceptibility Profile

To assess their safety as potential probiotic cultures, the susceptibility of the selected LAB strains to some highly clinically relevant antibiotics was evaluated using the Kirby-Bauer disk diffusion susceptibility test protocol using tailored commercial antibiotic disks (Bioscan, Sétif, Algeria). The antibiotics tested included Ampicillin (10 micrograms), Gentamicin (10 micrograms), Kanamycin (30 micrograms), Streptomycin (10 micrograms), Erythromycin (10 micrograms), Clindamycin (2 micrograms), Tetracycline (30 micrograms), Chloramphenicol (30 micrograms), and Vancomycin (30 micrograms). In brief, an overnight culture (18 h at 30 °C on MRS broth) of each LAB strain was adjusted to a 0.5 McFarland scale and inoculated on MRS agar. Commercial antibiotic disks were then placed on the surface of MRS plates [37]. After 24 h of incubation at 37 °C, the diameter of the clear zone around disks was measured (mm).

#### 2.2.7. Toxicity Assay (Hemolysis)

The hemolytic activity was tested by spreading overnight-grown LAB strains on Columbia agar media containing 5% (*w*/*v*) of human blood and incubating the plates at 30 °C for 48 h. The zones around the colonies determined (defined) the pattern of hemolysis on blood agar plates: β-hemolysis (clear halo) or α-hemolysis (green halo) and γ-hemolysis (no haloes) around the colonies [35].

#### 2.2.8. Tolerance to a Simulated GI Tract

For this experiment, simulated gastric juice was prepared to recreate the conditions of the human GI tract following the method presented by Oh et al. [40]. The gastric juice was prepared by suspending 3 mg/mL of pepsin in sterile PBS (phosphate-buffered saline, 0.2 M, pH 2.5). The solution was filtered through a 0.22 µm Millipore filter. To determine the in vitro survival of the isolates, LAB strains were centrifuged and resuspended in sterile saline solution (0.85% NaCl *w*/*v*). Each suspension was inoculated in the gastric juice at a final concentration of 10^8^ CFU/mL and incubated at 37 °C for four hours. Viable cell counts were assessed before (T0) and after incubation (T1) using the spread plate method on MRS agar [41]. The survival rate was determined using the previously mentioned formula.

#### 2.2.9. Auto-Aggregation and Co-Aggregation

Five milliliters of overnight culture, containing 10^8^ CFU/mL of the selected *L. plantarum* R12 and *W. cibaria* strains VR81 and LVT1, was vortexed for 10 s and incubated for 4 h at 37 °C. The absorbance of the supernatant was measured at t0 (ODt0) and after two hours of incubation (ODt) [42]. The auto-aggregation percentage was determined using the following formula: A = [1 − (ODt/ODt0)] × 100.

To assess co-aggregation, equal volumes (4 mL) of selected LAB strains and *L. innocua*, a close relative of *Listeria monocytogenes*, were mixed, vortexed for 10 s, and incubated at 37 °C. Absorbance values at 600 nm were measured at T0 and after 4 h of incubation for mixed (ODmix), single LAB (OD strain), and *L. innocua* culture (OD *L. innocua*). The percentage of co-aggregation was calculated as follows: co-aggregation (%) = [1 − OD mix/(OD strain + ODpathogen)/2] × 100.

#### 2.2.10. Adhesion Assay

Based on their probiotic and inhibitory activities, two strains of *W. cibaria* (VR81 and LVT1) were chosen to be further tested for their abilities to bind to the intestinal mucosal surfaces using Caco-2 cell lines, as previously described [42]. Briefly, Caco-2 cells were cultured in DMEM supplemented with 2 mM L-glutamine, 50 U/mL penicillin, 50 U/mL streptomycin, and 10% (*v*/*v*) heat-inactivated fetal bovine serum (FBS) (all from Gibco. Carlsbad, CA, USA) in 5% CO_2_ at 37 °C. Caco-2 cells were seeded in 96-well tissue culture plates (Gibco) and grown for 15 days in order to form monolayers. The investigated *W. cibaria* cultures were incubated with Caco-2 cells for one hour at 37 °C. Adherent bacteria were counted by plating after washing and compared to the total number of added bacterial CFU; thus, adhesion ability was expressed as a percentage, Adhesion% [(CFU) washed well/(CFU) unwashed well] × 100. The protocol was conducted in triplicates. *L. plantarum* 299 V served as a positive control [36]. 

### 2.3. Technological Characterization of the Selected Bioactive Strains

#### 2.3.1. Proteolytic Activity

To evaluate the extracellular proteolysis, LAB strains were cultivated on MRS media for 18 h, and then each strain was spotted (spot-inoculated) (10^8^ UFC/mL) on the surface of PCA media (Biolife Italiana S.R.L., Milan, Italy) supplemented with 3%, 5%, and 10% of skim milk. The plates were incubated at 30 °C for 72 h and were immerged with 1% HCL. The possession of proteolytic activity was reflected by the occurrence (appearance) of a clear hydrolysis zone around the spot; each zone was measured and expressed in mm. Analyses were carried out in triplicates [37].

#### 2.3.2. Lipolytic Activity

The lipolytic potential was examined by spotting 10 μL of LAB bacterial suspension on the surface of M17 media containing 1%, 3%, and 5% of tween 20. After an incubation of 72 h at 30 °C, the apparition of a halo around the spot defines the presence of lipolytic activity in LAB strains [43]. 

#### 2.3.3. Amylolytic Activity

In order to detect the presence of Amylase, the selected LAB strains were spotted on the surface of a modified MRS-starch (2%: 20 g/L starch and other components). After 24 h of incubation at 30 °C, the plates were exposed to iodine solution (Lugol) vapors and starch hydrolysis was revealed via the visualization of a clear zone around the LAB colonies, unlike blue-starched areas [44].

#### 2.3.4. Organic Acids Production

To identify and quantify the main organic acids produced by the selected LAB strains, the CFSs from an overnight culture on MRS broth were harvested (10,000× *g*, for 10 min) and analyzed using an HPLC Spectra System P1000 XR (Thermo Electron Corporation, Madison, WI, USA) [42].

#### 2.3.5. Exopolysaccharides Production and Quantification

The screening for EPS production using LAB strains involved streaking active pure cultures onto modified MRS agar. This modified agar was prepared by substituting glucose with various carbon sources, including sucrose (2%), lactose (2%), fructose (2%), maltose (10%), mannose (2%), and raffinose (2%). The cultures were incubated at 30 °C for 72 h. LAB strains that displayed slimy (mucoid) or ropy colony characteristics were identified as EPS producers.

To quantify EPS production, a fresh culture was inoculated into MRS broth supplemented with sucrose and incubated for 24 h. The cultures were then centrifuged at 6000 rpm for 20 min, and the resulting supernatants were transferred to empty flasks. Five volumes of cold ethanol were added to each flask, followed by another centrifugation at 6000 rpm at 4 °C for 20 min. After discarding the ethanol, the tubes containing the EPS pellet were dried at 60 °C for 24 h [45]. EPS yield was determined by calculating the weight difference between the empty and filled tubes containing the dried EPS pellet.

#### 2.3.6. Riboflavin Production and Direct Quantification 

To screen for riboflavin production using LAB strains, a preculture on MRS broth (early stationary phase) was centrifuged at 5000 rpm for 3 min at room temperature. The obtained pellet was washed twice with sterile saline solution to eliminate residual vitamin B and sedimented as above. The resulting pellet was resuspended in the same saline solution and used to inoculate CDRFM (Biolife Italiana S.R.L., Milan, Italy) at a ratio of (1:1000 *v*/*v*). The incubation was carried out under optimal growth conditions until the early stationary phase was reached. The strains that exhibited growth in the chemically defined media CDRFM were further cultivated in CDRFM supplemented with 10 mg/L of roseoflavin. Those demonstrating viability were exposed to increasing roseoflavin concentrations (50 mg, 100 mg, and 200 mg).

Briefly, bacterial cells from an overnight culture on MRS broth were washed twice and grown in CDRFM media for 24 h at optimal growth conditions. The riboflavin-producing strains were centrifuged at 11,000× *g* for 10 min at room temperature. An aliquot of 200 uL of the obtained supernatant of each LAB strain was transferred into a well of Nunc™ 96-Well Optical-Bottom Plates with a Polymer Base. Two replicates were performed for each strain. The fluorescence measurements were obtained by setting the BMG LABTECH reader (Ortenberg, Germany) at (λex ¼ 480 nm; λem ¼ 530 nm) at 30 °C. The riboflavin concentration was determined through the interpolation of fluorescence values on the calibration curve [46].

### 2.4. Cross-Over Food Application of Bioactive Strains on Quinoa Flour

#### 2.4.1. Survival Aptitude of Bioactive Strains against Abiotic Stress

In both environmental and in vivo conditions, LAB strains face a multitude of biotic and abiotic stresses, including acidity, thermal fluctuations, osmotic challenges, oxidative conditions, and others. These stressors significantly impact their activities and production efficiency during food fermentation. Numerous studies have demonstrated that pre-adaptation of LAB to a specific environmental stress can cross-protect against other stresses. For this purpose, we assessed the survival of *W. cibaria* VR81 and *L. plantarum* R12 under varying conditions. This included exposure to increasing concentrations of sucrose (0.2 M, 0.6 M, and 1 M) for osmotic stress [47], resistance to higher concentrations of hydrogen peroxide (62.5 µM, 125 µM, and 250 µM) after 4 h of exposure for oxidative stress [48], evaluating resistance and growth within a pH range of 3, 7, and 9 [49] and as well as testing the effect of higher temperature (65 °C) and lower temperature (4 °C) on bacterial growth. All tests were conducted in triplicate, with OD measurements at 600 nm using an ELISA plate reader (BioTek Instruments, Winooski, VT, USA) before and after exposure to the stress factor. A positive control involving growth in MRS media under optimum conditions was included for comparison.

#### 2.4.2. Application of *W. cibaria* VR81 and *L. plantarum* R12 as Primary Starters for Quinoa Beverage Design 

Two LAB strains belonging to *W. cibaria* VR81 and *L. plantarum* R12 were selected for their strong probiotic and biotechnological potential to produce spoonable plant-based yogurt. These strains serve as the starter culture, steering the bacterial fermentation of quinoa flour *Chenopodium quinoa*, commonly referred to as quinoa, stands out as a gluten-free pseudocereal recognized as one of the finest vegetal protein sources [50], comprising 13.1–16.7% of edible matter. It is rich in protein, dietary fiber (7.0–11.7% of edible matter), and thiamine, folic acid, vitamins C, B6, E, and pantothenic acid. Additionally, it offers essential fatty acids, with 38.9–57% of linoleic acid, 24.0–27.7% of oleic acid, and 4% of α-linolenic acid. Quinoa further provides essential dietary minerals such as calcium, iron, magnesium, and potassium, along with bioactive components [51]. One variety of quinoa, Rosada de Huancayo, was purchased online, washed until the complete removal of saponins, and subsequently dried at 60 °C for a minimum of 8 h. The resulting dried quinoa was finely ground to obtain the corresponding flour, which was then stored at 4 °C. Our study involved two fermentation processes: one with a co-culture of *W. cibaria* VR81 and *L. plantarum* R12 fermenting quinoa, and the other with each LAB in a single culture. Following the method outlined by Väkeväinen et al. [24], quinoa flour was mixed with water (20%) and incubated for 10 min at 80 °C for gelatinization. After cooling at room temperature, each LAB (*W. cibaria*, *L. plantarum*) was separately inoculated at a concentration of 1% in the quinoa mixture for monitoring a single culture. Mixed cultures were prepared by simultaneously inoculating quinoa with *W. cibaria* VR81 and *L. plantarum* R12 at a final concentration of 10^7^ UFC/mL. The samples were fermented at 30 °C for approximately five hours until reaching a pH < 4, then stored at 4 °C for 28 days. In addition, we conducted a spontaneous fermentation of quinoa following the same conditions to observe the influence of indigenous microflora and a negative control involved quinoa without fermentation. The pH was measured at T0, then every two hours for 24 h, and subsequently on a daily basis for up to 28 days of storage.

#### 2.4.3. Sensory Quality

A sensory evaluation was conducted on the quinoa beverage fermented with various inoculation strategies, which included co-inoculation with *W. cibaria* and *L. plantarum*, single inoculation with either *L. plantarum* or *W. cibaria*, and an uninoculated control. This evaluation was performed by a panel of five trained panelists who assessed the sensory characteristics at all experimental time points. The samples were stored at 4 °C until the analysis. Each panelist was provided with three samples from each treatment, presented in lidded containers to preserve the aroma. To ensure objectivity in the evaluation, the samples were randomly coded with three-digit numbers, masking their treatment identities. The panelists evaluated several sensory attributes, including color, odor, off-odor, visual quality, and overall appearance. A hedonic scale, featuring five pictures, was employed for scoring, with values ranging from 1 (indicating poor quality) to 5 (indicating optimal characteristics).

#### 2.4.4. Quantification of Riboflavin in Fermented Quinoa Product

To assess riboflavin production in the fermented quinoa by LAB overproducers, we followed the protocol described by Jakobsen [52]. Initially, A volume of 25 mL of HCl 0.1 M (prepared by dilution in water milli Q) was added to 5 g of the fermented quinoa (co-culture, and single culture with *L. plantarum* R12 and *W. cibaria* VR81) then the mixture was subjected to a heat treatment at 121 °C for 30 min. After cooling to room temperature, the pH was adjusted to 4.5 using a 4 M sodium acetate solution. A volume of 5.2 mL of an enzymatic solution consisting of 4.5 mL of α-amylase, 0.2 mL of papain, and 0.5 mL of 1% reduced glutathione, was added to the quinoa mixture. The incubation was performed at 45 °C for 18 h in a shaker–incubator (Argolab, Carpi, Italy). 

After the incubation period, the digested mass was transferred to a falcon, and a volume of HCl 0.01 was added to reach a final volume of 50 mL. The mixture was then centrifuged at 8000× *g* for 10 min, and the obtained supernatant was initially filtered through 4 layers of gauze, followed by the filtration of the entire volume through a 0.22 µm Millipore filter. An aliquot of 0.2 mL of the obtained supernatants from the parental and mutant LAB overproducers was filled into the 96-well plate. The fluorescence measurements were obtained by setting the BMG LABTECH reader at (λex ¼ 480 nm; λem ¼ 530 nm) at 30 °C. The riboflavin concentration was determined through the interpolation of fluorescence values on the calibration curve.

#### 2.4.5. Application of *W. cibaria* VR81 and *L. plantarum* R12 as Bioprotector Culture on Quinoa Flour

To assess the in vivo antimicrobial potential of the selected LAB strains against *L. innocua*, *W. cibaria* VR81 and *L. plantarum* R12 were inoculated separately and in co-culture in the quinoa mixture along with *L. innocua* at a ratio of 1% each. The fermentation was conducted at 30 °C for 5 h, followed by storage of the quinoa beverage at 4 °C for 28 days. After 5 h of incubation, the fermented quinoa samples were serially diluted (10^8^). The spoilage and pathogenic microorganisms present in the final fermented quinoa were assessed by counting each flora on its respective selective media at the following time points: T0, T1, T7, T14, and T28. Enumeration included FAMT on PCA agar, molds and fungi on PDA (Biolife, Italiana S.R.L., Milan, Italy), coliforms on VRBG (Biolife, Italiana S.R.L., Milan, Italy) agar, *Staphylococcus* spp. on Chapman (Biolife, Italiana S.R.L., Milan, Italy) agar, and *L. innocua* on Palcam (Biolife, Italiana S.R.L., Milan, Italy) agar. The experiment was carried out in triplicate. 

To assess the dominance of the starter cultures (*W. cibaria* VR81 and *L. plantarum* R12) in the fermented quinoa, the viable cells were enumerated using MRS + 2% sucrose. After 24 h of incubation, the presence of *W. cibaria* was indicated by the count of slimy colonies as they are EPS producers, while the presence of *L. plantarum* was indicated by the presence of small opaque colonies. The dominance of *W. cibaria* VR81 and *L. plantarum* R12 was also evaluated in the fermentation of other plant-based milk products, serving as controls, including soy milk, coconut milk, oat milk, and skim milk.

### 2.5. Statistical Analysis 

All of the assays were performed in triplicate, and statistical analysis was conducted using the XLSTAT package (version 2019.3.2 for Windows). Before performing the analysis of variance (one-way ANOVA, two-way ANOVA) with Tukey test for significant differences (*p* < 0.05), normal distribution of the data was verified using the Kolmogorov–Smirnov test.

## 3. Results

### 3.1. Isolation and General Characterization of LAB

#### 3.1.1. Isolation and Preliminary Screening

A total number of 250 potential LAB isolates were obtained from diverse Algerian dairy products (i.e., raw milk, jben, and leben; all from camel and goat) from different geographical origins. The preliminary phenotypical identification revealed significant differences in the diversity and distribution of LAB strains among the tested dairy samples (Table S1, Supplementary Materials). Based on this characterization (Section 3.1.2), the predominant presence of *Enterococcus* and *Leuconostoc* genera was observed in both camel and goat milk samples, whereas *Weissella* and lactobacilli were less prevalent. In contrast, *Jben* samples exhibited a substantial abundance of lactobacilli along with the *Enterococcus* genus. During the pre-selection phase, 12 LAB isolates/strains isolated from different raw and fermented goat and camel milk samples displayed a strong ability to inhibit at least two indicator foodborne pathogens, as assessed by the agar disk diffusion assay. After genotypic characterization (Section 3.1.3), the strains were found to belong to *L. plantarum* and *W. cibaria* species. A strong antagonism was noticed against *P. aeruginosa*, *S. aureus* and *L. innocua* among the tested LAB isolates with inhibition diameter zones ranging from 10 mm to 25 mm (Table 1).

Notably, *L. plantarum* exhibited stronger antagonism against *L. innocua*, with strain R12 revealing the largest inhibition zone of 25 mm. Conversely, *W. cibaria* showed broader inhibition against *P. aeruginosa*, with strain ME9 also demonstrating a significant inhibition zone of 25 mm. Both species, however, exhibited weaker antagonistic effects against *S. enterica*, with strain R17 showing the maximum inhibition zone of 16.66 mm. There are some species-dependent trends, suggesting that a combination of the LAB species could target specific pathogens more effectively.

#### 3.1.2. Physiological and Biochemical Characterization of the Selected LAB Strains

The growth ability of the strains was assessed across a range of temperatures. All strains exhibited rapid growth at 15 °C, 37 °C, and 45 °C, reaching their peak optical density within 18 h of incubation. However, at 4 °C, their growth was limited even after 48 h of incubation. Furthermore, to assess their tolerance to high temperatures, the strains underwent exposure to 55 °C and 63 °C for 30 min. At both temperatures, all the strains displayed a high level of resistance, maintaining a viability of over 90%. After exposure to a pH of 4.8 and 6.8, all of the tested LAB strains displayed robust growth within this range. Similarly, the LAB strains also exhibited strong growth at 3% and 6.8% of NaCl (Table S1, Supplementary Materials). Concerning carbohydrate fermentation, the results of the different strains are represented in Table S2 of the Supplementary Materials. Both *W. cibaria* (VR81, LVT1) and *L. plantarum* strain R12 were able to ferment glucose, fructose, sucrose and 5-keto-gluconate. Additionally, *W. cibaria* strains VR81 and LVT1 ferment galactose, maltose, mannitol, mannose, raffinose, ribose, trehalose, esculin, galactose, and lactose. Finally, *L. plantarum* R12 was the only strain able to ferment melibiose.

#### 3.1.3. Genotypic Characterization of Isolates

The 16S rRNA sequences of the 12 selected isolates were edited using BioEdit and analyzed with MEGA software, version 11 (64-bits), and compared to GenBank via BLAST. The BLAST results revealed that out of the twelve identified strains, 58.3% (seven strains) of the antimicrobial-producing strains belonged to *Weissella cibaria*, with more than 99% homology, and 41.7% (five strains) belonged to *Lactiplantibacillus plantarum*, with a similarity > 99%.

### 3.2. Probiotic Evaluation

#### 3.2.1. Screening for Antimicrobial Activity 

**Figure 1:**
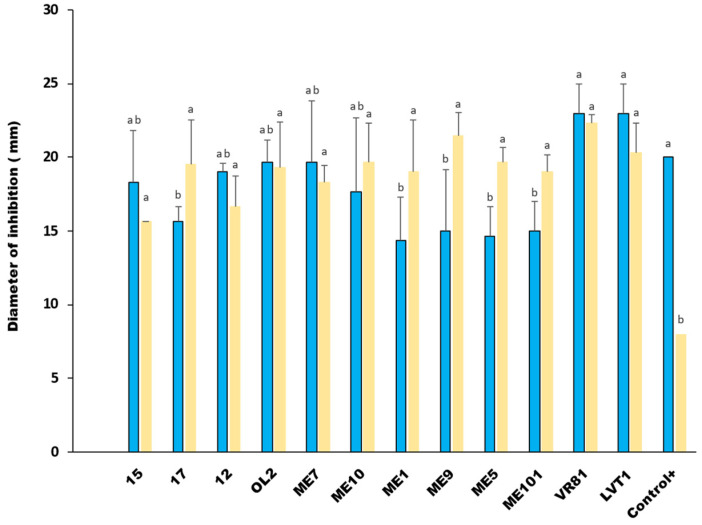
Diameter of the inhibition zones against *P. aeruginosa* (Yellow bars) and against *L. innocua* (blue bars) using the CFS of the isolated LAB via agar well diffusion assay (student test). Error bars indicate the standard deviation of triplicate experiments. The same letters indicate no significant differences (*p* < 0.05). Different letters indicate significant differences (*p* < 0.05), level a > b.

Twelve LAB strains, identified as *W. cibaria* and *L. plantarum*, and exhibiting the broadest inhibition zones, underwent further screening using the agar well diffusion assay instead of agar disk diffusion assay. The results revealed that the inhibitory effect of 50% of the selected isolates considerably decreased after testing their CFS against the same indicator pathogens, whereas the remaining strains maintained a strong inhibitory activity against *P. aeruginosa* and *L. innocua*, with diameter zones ranging from 10 mm to 25 mm (Figure 1). The largest diameters were detected with *W. cibaria* strains VR81 and LVT1 along with *L. plantarum* OL2 and R12 towards *L. innocua*. Concerning *S. enterica*, the CFS of all LAB strains showed limited to no inhibitory effect on its growth.

#### 3.2.2. Determination of the Proteinaceous Nature of the Inhibitory Substance 

**Figure 2:**
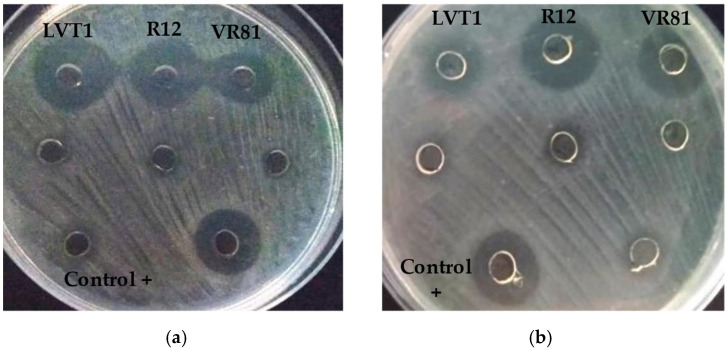
Inhibitory effect of NCFS and NCFS + proteolytic enzyme from *W. cibaria* VR81, LVT1 and *L. plantarum* R12 against *P. aeruginosa* (**a**); inhibitory effect of NCFS and NCFS + enzyme from *W. cibaria* VR81, LVT1 and *L. plantarum* R12 against *L. innocua* (**b**). The upper wells contain the NCFS of LAB strains; the lower wells contain the NCFS treated with proteolytic enzyme. The positive control used in the experiment is represented by strain B7 (*Leuconostoc mesenteroides*), serving as a reference for bacteriocin production.

The proteinaceous nature of the inhibitory agent was evaluated by treating the NCFS (neutralized cell-free supernatant) of the twelve LAB candidates with proteolytic enzymes trypsin and chymotrypsin. The results showed variations among the tested strains; notably, a complete loss of the inhibitory effect was observed against both *P. aeruginosa* and *L. innocua* with the strains LVT1, R12, and VRB81. Meanwhile, the antimicrobial activity remained stable with high inhibition zones for the non-treated neutralized CFS from the same strains (Figure 2).

However, the results of the neutralized cell-free supernatant of the remaining nine strains showed minimal to no inhibition against the indicator microorganisms, suggesting that their inhibitory potential was mainly due to organic acid production. According to these results, two strains belonging to *W. cibaria* (VR81-LVT1) and one strain belonging to *L. plantarum* (R12) were selected for further studies.

#### 3.2.3. Effect of Heat Treatment, pH, and Chemical Detergents on the Bacteriocin Activity

The antimicrobial action of the bacteriocin-like substance produced by the *W. cibaria* VR81, LVT1, and *L. plantarum* R12 strains remained stable and effective across a wide pH range from 3 to 6.8. However, a distinct alteration was observed at pH 9, where the diameter of the inhibition zone noticeably decreased, eventually reaching zero. Regarding stability at high temperatures, the effectiveness of the bacteriocin-like substances produced by *W. cibaria* strains VR81, LVT1, and *L. plantarum* R12 remained consistent following treatments at 60 °C, 70 °C, 80 °C, and 90 °C. However, a complete loss of inhibition was observed at 100 °C and 120 °C. The antimicrobial activity of cell-free supernatant (CFS) from all strains decreased after treatment with urea, while the addition of Tween 80 resulted in an increased inhibition spectrum for all the tested strains (Table 2).

#### 3.2.4. Acid and Bile Salt Tolerance

**Figure 3:**
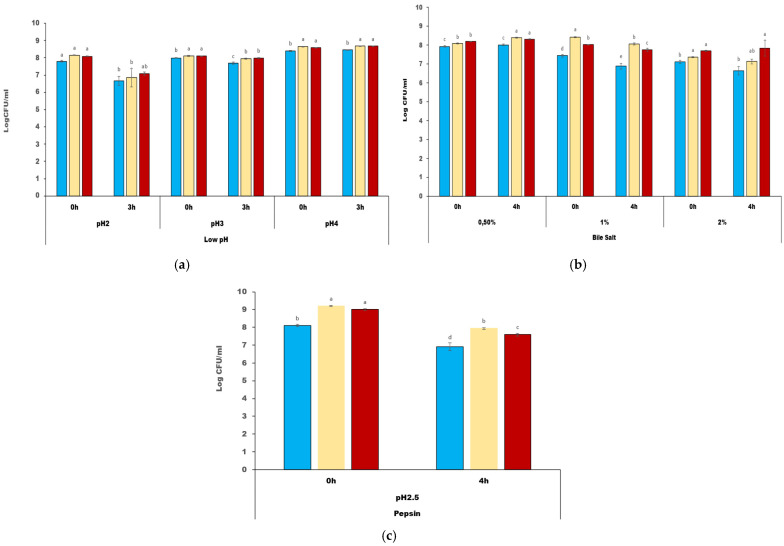
Tolerance of *W. cibaria* VR81 (yellow bars), LVT1 (blue bars), and *L. plantarum* R12 (red bars) to low pH (**a**), to bile salt (**b**), and to pepsin (**c**). The same letters indicate no significant differences (*p* < 0.05). Different letters indicate significant differences (*p* < 0.05), with the levels ranked as a > b > c > d. Error bars represent the standard deviation of three independent experiments.

While variability was noted among the tested LAB isolates, the findings indicate a significant tolerance to low pH across all strains, with R12, VR81, and LVT1 exhibiting survival rates of over 70% and over 60% at pH 2, respectively. In particular, *L. plantarum* R12 emerged as the most robust strain, demonstrating the highest viability with over 70% survival rate at both pH 2 and pH 3, along with a growth increase of 0.08 log units at pH 4 (Figure 3). In the assessment of bile salt tolerance, all strains showed a notable increase in growth in the presence of 0.5% oxgall, with increases of 0.31 log, 0.2 log, and 0.09 log units for strains VR81, R12, and LVT1, respectively (Figure 3).

Notably, strains R12 and VR81 demonstrated the highest resistance among the tested strains in the presence of 2% oxgall. *W. cibaria* VR81 displayed 97% survival ability, while *L. plantarum* R12 showed a growth increase of 0.15 LogCFU/mL. Additionally, no bile salt hydrolysis was observed among the LAB strains.

#### 3.2.5. Resistance to Pepsin and Hemolysis

In the evaluation of resistance to pepsin, the tested strains demonstrated significant resistance levels after 4 h of exposure to 3 mg/mL of pepsin. *W. cibaria* VR81 showed the highest resistance, with 86% survival ability, followed closely by *L. plantarum* R12, with 84% survival. *W. cibaria* (LVT1) showed the least survival potential, 76%, as shown in Figure 3. Moreover, none of the tested strains exhibited any hemolytic activity. 

#### 3.2.6. Adhesion to Caco-2 Cells

Adhesion to the intestinal mucosa is crucial for a probiotic strain; it acts as a barrier against enteropathogenic bacteria, preventing their adhesion to intestinal cells and creating a competitive environment for nutrients [53]. The adhesion test results revealed that *W. cibaria* strains LVT1 and VR81 have strong adhesion capacities to Caco-2 cell lines. In fact, their adhesion percentage was higher than the positive control *L. plantarum* 299 (2.51%), with a percentage of 7.09% for VR81 and 6.66% for LVT1.

#### 3.2.7. Evaluation of Auto-Aggregation and Co-Aggregation

The ability of a probiotic strain to auto-aggregate is a crucial factor influencing its adherence to the oral cavity, gastrointestinal tract, and urogenital tract [54]. The auto-aggregation results revealed variations among the tested strains, showing a notable dependence on the incubation period. Notably, *W. cibaria* VR81 demonstrated the greatest auto-aggregation ability, with 37.8% after four hours and 65.7% after 24 h of incubation. This was followed closely by *L. plantarum* R12, which exhibited 27% and 55.6% auto-aggregation at 4 and 24 h, respectively. The lowest auto-aggregation rate, observed after 4 h and 24 h of incubation, was noted for *W. cibaria* LVT1, with 21% and 48% rates, respectively, as shown in Table 3. 

The results of co-aggregation with *L. innocua* indicate consistent adherence to the tested LAB strains. *W. cibaria* VR81 demonstrated stable performance, with adherence percentages of 21.0% and 21.1% after 4 and 24 h of incubation. Similarly, *L. plantarum* R12 displayed significant adherence, with percentages of 19.1% and 18.6% at 4 and 24 h, respectively. In contrast, LVT1 exhibited the lowest adherence percentage, with 16.5% and 15.1% at 4 and 24 h, respectively, as shown in Table 4.

#### 3.2.8. Antibiotic Susceptibility

In the absence of defined antibiotic susceptibility breakpoints for *Weissella* via EFSA in 2012, the interpretation of results relied on the cut-off values designated for *Leuconostoc* strains, given their close taxonomic relationship [55]. The disk diffusion method evaluated the phenotypic susceptibility of *W. cibaria* VR81, LVT1 and *L. plantarum* R12 to nine antibiotics. As shown in Table 5, the three bioactive strains exhibited resistance to Kanamycin and Vancomycin while demonstrating susceptibility to Gentamycin, Chloramphenicol, and Clindamycin. Significant variations were noted toward four antibiotics: *W. cibaria* VR81 showed intermediate susceptibility to Ampicillin compared to LVT1 and *L. plantarum*’s sensitivity. Additionally, both *W. cibaria* strains VR81 and LVT1 were sensitive to Streptomycin, in contrast to *L. plantarum* resistance. LVT1 showed resistance to Tetracycline and susceptibility to Erythromycin, in contrast to the response observed with *W. cibaria* VR81 and *L. plantarum* R12.

### 3.3. Technological Characteristics of the Bioactive Strains

#### 3.3.1. Proteolytic, Amylolytic and Lipolytic Activity 

In the dairy industry, proteolytic and lipolytic properties play a key role in ensuring successful fermentation; the released peptides and amino acids create aroma, texture, and taste in the final product [56]. Regarding enzymatic activity, the twelve LAB strains displayed the ability to degrade casein in skim milk at concentrations of 3%, 5%, and 10%, as indicated by the presence of clear halos around their colonies. However, variations were notable among the LAB strains, with strains VR81 and R12 demonstrating the highest proteolytic values (Table S3, Supplementary Materials). Conversely, weak lipolytic activity was only observed in *L. plantarum* R12 strain, while none of the strains exhibited amylolytic activity.

#### 3.3.2. Acidifying Activity, Milk Coagulation

The acidifying activity of LAB plays a pivotal role in selecting starter cultures, as it guides fermentation by reducing the pH, enhancing food safety, and contributing to the desired taste and texture of the final product. The acidifying activity results within 24 h of growth in skim milk and various plant-based beverages showed significant variations among the different matrices. Both skim and coconut milk exhibited a slight pH decrease, dropping by 1.34 ± 0.14 to 1.40 ± 0.24 units after 24 h of incubation with both *W. cibaria* and *L. plantarum* strains. In contrast, the acidification in other plant-based beverages was more effective, with rapid pH decreases reaching 3.64 ± 0.12, 4.26 ± 0.41, and 4.78 ± 0.16 units for the quinoa beverage, oat milk, and soy milk, respectively, when *L. plantarum* R12 was used as the starter culture, *W. cibaria* VR81 exhibited similar results, with pH decreases of 3.54 ± 0.22, 4.46 ± 0.15, and 4.68 ± 0.12 units, respectively (Figure S1, Supplementary Materials). Regarding milk coagulation, only one strain belonging to *L. plantarum* R12 was able to partially coagulate skim milk after 24 h of incubation. However, among the other matrices, the quinoa beverage and soy milk showed the best results, with complete coagulation occurring after 16 h when *W. cibaria* VR81 and *L. plantarum* R12 were used as starter cultures.

#### 3.3.3. Identification of Organic Acids Produced by LAB Strains via HPLC

Organic acids produced by LAB play a crucial role in food preservation and flavor enhancement, contributing to the safety and sensory qualities of fermented products. The heterofermentative profile of the twelve *W. cibaria* and *L. plantarum* strains was confirmed using HPLC analysis (Table 6), revealing a diverse production of organic acids. Acetic acid was the most abundant, with *W. cibaria* ME1 producing the highest concentration at 44 g/L, while *W. cibaria* ME5 exhibited the lowest at 26.15 g/L. Lactic acid was the second most dominant, with the *L. plantarum* ME10 strain achieving a peak concentration of 11.75 g/L, whereas the lowest level was observed in *W. cibaria* ME7, at 4.64 g/L. The strains also produced significant amounts of tartaric and malic acids. *W. cibaria* VR81 produced the highest concentration of tartaric acid at 2.39 g/L, while *L. plantarum* ME10 had the highest malic acid production at 3.54 g/L. Ascorbic acid was also synthesized by all strains, with *L. plantarum* R12 reaching the highest level of 0.74 g/L. Regarding succinic and fumaric acids, notable variations were observed among the strains. The highest concentration of succinic acid was recorded in *L. plantarum* R17 at 11.24 g/L. Fumaric acid production was limited to only a few strains, with *L. plantarum* R12 showing the highest concentration at 13.35 mg/L, while *W. cibaria* ME1 produced the lowest at 2.26 mg/L.

#### 3.3.4. Exopolysaccharide Production and Quantification

Exopolysaccharide (EPS) production plays a crucial role in enhancing the probiotic qualities of presumed probiotic bacteria. In our study, all *W. cibaria* and one *L. plantarum* strain R15 exhibited strong EPS production, characterized by the formation of large, viscous colonies, when sucrose was used as the carbon/sugar source. Notably, one *L. plantarum* R12 strain displayed weak EPS production when maltose was used as the sugar source. No EPS production was observed among the strains when other sugar sources were employed. EPS quantification of strains with a positive exopolysaccharide (EPS) phenotype using sucrose as the sugar source revealed variations in production. *W. cibaria* strain VR81 produced the highest amount (4.7 mg/mL), followed by LVT1 (3.6 mg/mL), while the lowest production was noted in *L. plantarum* R15 strain (1.08 mg/mL) after 24 h of incubation (Table S4, Supplementary Materials).

#### 3.3.5. Screening for Riboflavin Production by LAB Strains

**Figure 4:**
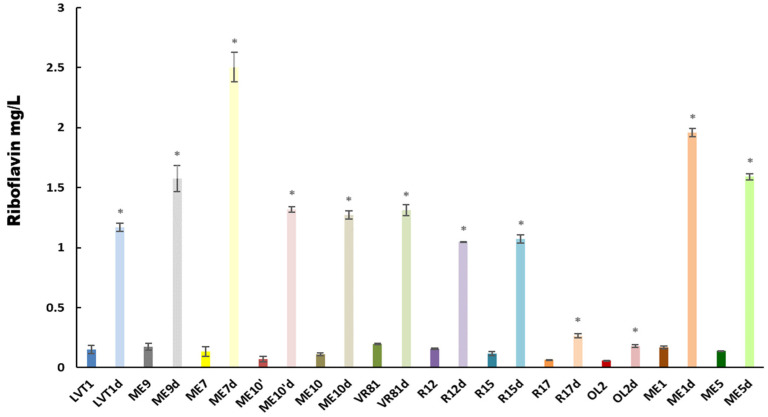
Quantification of riboflavin levels in the CFS (cell-free supernatant) of the twelve wild-type *W cibaria* and *L plantarum* strains and their mutants (strain code + d) via fluorescence analysis. Dark bars indicate the wild type, and light bars indicate the mutant. Error bars indicate the standard deviation of three experiments. Comparing each parental/mutant strain couple, *: *p* < 0.05.

Lactic acid bacteria (LAB) are increasingly recognized for their ability to synthesize B-group vitamins, particularly riboflavin, during fermentation. This ability enhances the nutritional profile of fermented foods, underscoring the significance of LAB strains in developing nutritionally enriched products. To identify riboflavin-overproducing strains with potential applications in the production of a fermented quinoa beverage enriched in riboflavin, twelve strains, belonging to *W. cibaria* and *L. plantarum*, were subjected to increasing concentrations of roseoflavin. The obtained spontaneous mutant strains (LVT1d, ME9d, ME7d, ME10′d, ME10d, VR81d, R12d, R15d, ME1d, and ME5d)) were resistant to the riboflavin homolog up to a concentration of 100 mg/L. Quantification of riboflavin levels in the cell-free supernatant (CFS) of all mutant and parental strains of *W. cibaria* and *L. plantarum* revealed that the mutant strains produced significantly higher levels of riboflavin than their corresponding parental strains. The highest production level was observed with *W. cibaria* ME7d, reaching 2.505 mg/L, followed by ME1d, which exhibited a lower level of 1.95 mg/L. *L. plantarum* R112 showed the lowest production, with 1.07 mg/L (Figure 4).

### 3.4. Cross-Over Food Application of Bioactive Strains W. cibaria VR81 and L. plantarum R12 on Quinoa Flour 

#### 3.4.1. Resistance of LAB to Abiotic Stressors

LAB face various challenges, including abiotic and biotic stresses (e.g., acidic, thermal, osmotic, oxidative and other stresses); these factors significantly impact their metabolic activities, hence affecting food fermentation efficiency [57]. In our study, we evaluated the resistance of *W. cibaria* VR81 and *L. plantarum* R12 to various abiotic stressors in the context of fermenting quinoa flour. These strains were chosen based on their high probiotic potential and good technological properties, including the production of bacteriocin-like substances, resistance to low pH and bile salts, as well as their ability to produce high amounts of exopolysaccharides (EPSs) and riboflavin. By evaluating their performance under abiotic stress conditions, our objective was to determine their suitability as starter cultures for fermenting quinoa seeds. This is essential as achieving successful fermentation and ensuring product quality preservation rely on the presence of robust starter cultures that maintain their viability in the final product. The addition of increasing levels of hydrogen peroxide resulted in a notable reduction in bacterial growth for both *L. plantarum* R12 and *W. cibaria* VR81 when compared to growth without hydrogen peroxide. *L. plantarum* R12 exhibited a better survival rate of 39% at a hydrogen peroxide concentration of 62.5 µM, whereas *W. cibaria* displayed a lower survival rate of 19% at the same concentration. Only slight changes in cell viability were observed at higher concentrations (125 mM and 250 mM hydrogen peroxide), with *L. plantarum* R12 strain showing a modest decrease to 33% and 31%, and *W. cibaria* strain VR81 showing a slight increase to 20% and 22%, respectively. Similar to oxidative stress, higher concentrations of sucrose, lower pH levels, and extreme temperatures decreased the bacterial load of the two selected strains. The survival rate in the presence of 1 M sucrose was significantly higher with *W. cibaria* strain VR81, reaching 41%, compared to *L. plantarum* strain R12, which achieved a lower rate of 25%. Regarding the resistance of *W. cibaria* and *L. plantarum* to extreme temperatures, survival rates exceeded 8% at high temperatures, while at 4 °C, survival rates dropped below 5% for both strains Table 7. 

#### 3.4.2. Application of *W. cibaria* and *L. plantarum* as Primary Starters for Quinoa Beverage Design, Separately and in Co-Inoculation

**Figure 5:**
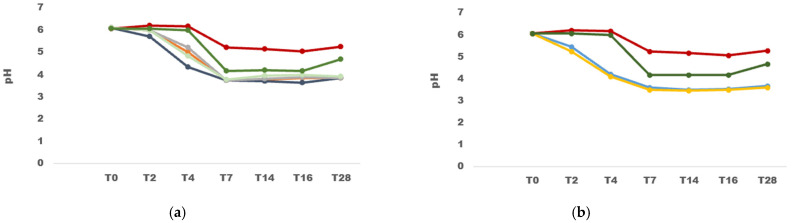
(**a**) pH assessment of fermented quinoa over time using single cultures of LAB (*L. plantarum*, *W. cibaria*) as the starter against *L. innocua*. The orange curve represents quinoa inoculated with *L. plantarum*; the dark blue curve represents quinoa inoculated with *W. cibaria*; the gray curve represents quinoa inoculated with *L. plantarum* + *L. innocua*; the light green curve represents quinoa inoculated with *W. cibaria* + *L. innocua*; the red curve represents quinoa fermented by spontaneous flora; the dark green curve represents quinoa inoculated with *L. innocua*. (**b**) pH assessment of fermented quinoa over time using a co-culture of LAB (*W. cibaria* + *L. plantarum*) as the starter against *L. innocua*. The light blue curve represents the co-culture of *W. cibaria* + *L. plantarum*; the red curve represents quinoa fermented by spontaneous flora; the yellow curve represents quinoa inoculated with both LAB (*W. cibaria* + *L. plantarum*) and *L. innocua*; the dark green curve represents quinoa inoculated with *L. innocua.* Error bars indicate the standard deviation of triplicate experiments.

During the fermentation process of quinoa flour in both single and mixed cultures, pH levels were measured before and after LAB inoculation. A distinct coagulum formation was observed after five hours of incubation at 30 °C. Notably, a significant pH decrease to 3.97 was observed in the co-culture (mixed) fermentation after 5 h of incubation, while the single fermentations with *W. cibaria* VR81 and *L. plantarum* R12 individually reached a pH below 4 only after 6 h of incubation. In the spontaneous fermentation of quinoa and quinoa inoculated with *L. innocua*, the pH reached 5.05 and pH 4.19, respectively, after 16 h of incubation. Higher acidification was noted when LAB strains were co-cultured with, reaching pH < 4 after 6 h of incubation (Figure 5).

#### 3.4.3. Sensory Evaluation

**Figure 6:**
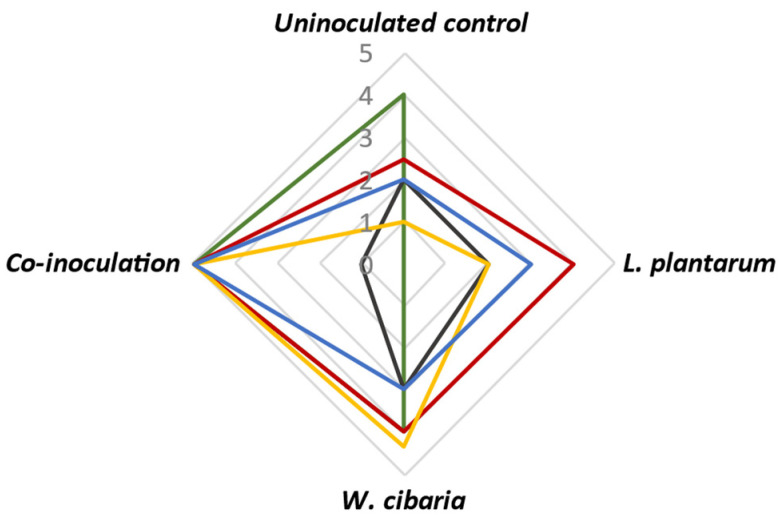
Sensory evaluation of a functional quinoa-based beverage fermented with co-inoculation (*L. plantarum* + *W. cibaria*), single inoculation (*L. plantarum* or *W. cibaria*), and uninoculated control after 28 days of storage at 4 °C: color-coded representation of attributes (green for color, red for odor, black for off-odor, yellow for visual appearance, and blue for overall acceptance).

The sensory evaluation of the quinoa beverage revealed variations among the different inoculation strategies (Figure 6). The odor of the quinoa beverage fermented with the co-inoculation of *W. cibaria* and *L. plantarum* exhibited a desirable spicy aroma similar to cucumber pickles. In contrast, the beverages fermented with a single culture of *L. plantarum* or *W. cibaria* presented a sour aroma, while the uninoculated control displayed a faint aroma comparable to roasted grains. Visually, the quinoa beverage inoculated with the co-culture of *W. cibaria* and *L. plantarum*, as well as the single inoculation of *W. cibaria*, exhibited the most favorable results, displaying a semi-solid texture with fine bubbles, which could be attributed to CO_2_ production. In contrast, the beverage inoculated with *L. plantarum* and the uninoculated control exhibited a more liquid consistency without CO_2_ production. 

#### 3.4.4. Quantification of Riboflavin in Fermented Quinoa

**Figure 7:**
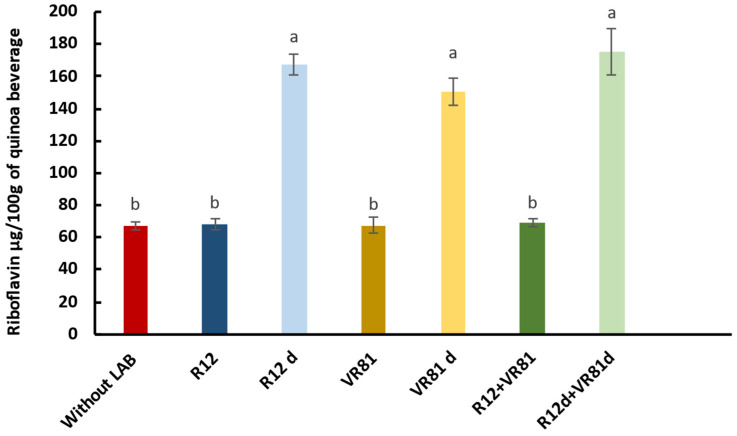
Quantification of riboflavin content in quinoa beverage fermented with the parental strains of *W. cibaria* VR81 and *L. plantarum* R12 and their mutants (strain code + d) by fluorescence analysis. Error bars indicate the standard deviation of three experiments. The same letters indicate no significant differences (*p* < 0.05). Different letters indicate significant differences (*p* < 0.05), with the levels ranked as a > b.

The application of the two selected riboflavin-overproducing LAB strains (VR81d, R12d) in the production of quinoa beverages revealed significant findings. Various conditions were tested to compare riboflavin production between wild-type and mutant strains in the quinoa matrix. The results showed that riboflavin levels in quinoa fermented with the parental strains did not significantly differ from the unfermented quinoa (quinoa without LAB). However, when quinoa was fermented using the mutant strains (R12d and VR81d), both individually and in co-culture, there was a considerable increase in riboflavin levels. The highest riboflavin production was observed in the co-culture of mutant strains R12d+ VR81d, reaching 175.41 µg/100 g, a 2.54-fold increase compared to the parental co-culture (R12 + VR81). This was followed by the single-culture fermentation of *L. plantarum* R12d, where riboflavin concentration attained 167.04 µg/100 g, representing a 2.45-fold increase compared to the parental strain R12. Finally, the *W. cibaria* mutant strain VR81d showed a 2.24-fold increase in riboflavin production compared to its corresponding parental strain VR81, as shown in Figure 7. 

These results demonstrate that the selected mutant LAB strains (R12d and VR81d) significantly enriched the riboflavin content of the fermented quinoa beverage compared to their parental strains, confirming our previous findings (Section 3.3.4) where the mutant strains produced significantly higher riboflavin levels in CDM medium.

#### 3.4.5. Application of *W. cibaria* and *L. plantarum* as Bio-Protective Culture on Quinoa Beverage

**Figure 8:**
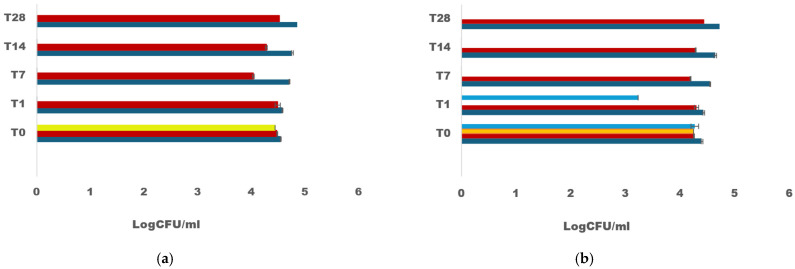
(**a**) Growth evolution of *L. innocua* in fermented quinoa during 28 days of fermentation using a co-culture of LAB (*W. cibaria* + *L. plantarum*) as the starter. The yellow curve represents the count of *L. innocua* in fermented quinoa using a co-culture of LAB (*L. plantarum* + *W. cibaria*); the red curve represents the count of *L. innocua* in fermented quinoa using the indigenous microflora; the dark blue curve represents the count of *L.innocua* in quinoa without fermentation (positive control). (**b**) Growth evolution of *L. innocua* in fermented quinoa during 28 days of storage using single cultures of LAB (*L. plantarum* and *W. cibaria* separately) as starters. The orange curve represents the count of *L. innocua* in fermented quinoa using *L. plantarum* as the starter culture; the light blue curve represents the count of *L. innocua* in fermented quinoa using *W. cibaria* as the starter culture; the red curve represents the count of *L. innocua* in fermented quinoa using the indigenous microflora; and the dark blue curve represents the count of *L.innocua* in quinoa without fermentation (positive control). Error bars indicate the standard deviation of triplicate experiments.

Regarding the hygienic control of quinoa, the results are represented in Figure 8. In the mixed culture, the LAB strains (*W. cibaria* VR81 + *L. plantarum* R12) exhibited the complete inhibition of the pathogen *L. innocua*, with 0% growth on Palcam media starting from T1 (24 h). 

In single-culture fermentation (T1), *L. plantarum* R12 demonstrated the complete inhibition of *L. innocua*, whereas *W. cibaria* VR81 showed partial inhibition with a microbial load decrease of 1.10 log CFU. However, from T7 to T28, both *L. plantarum* and *W. cibaria* completely inhibited the growth of *L. innocua*. The growth of fungi on PDA, of *Staphylococcus* sp. on Chapman and coliforms on VRBL was completely inhibited with mixed and single culture for up to 28 days.

#### 3.4.6. Dominance of LAB in the Fermented Quinoa

**Figure 9:**
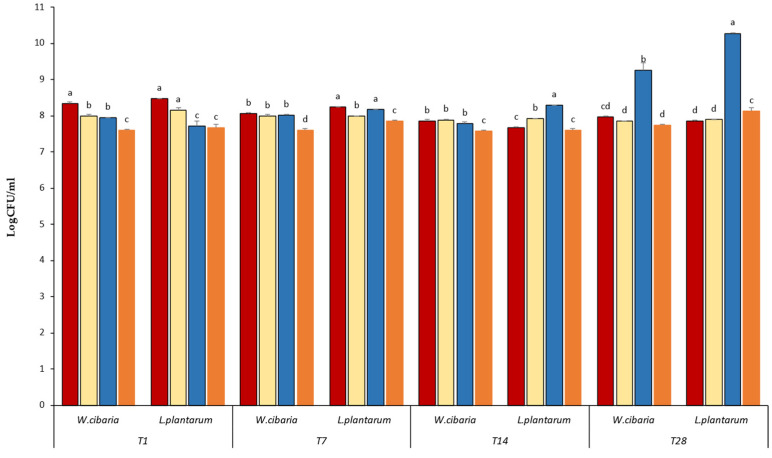
Dominance of *W. cibaria* VR81 and *L. plantarum* R12 in co-cultured fermentation of various plant-based milks: a quantitative analysis of LAB counts in quinoa beverage (red bars), soy milk (yellow bars), coconut milk (blue bars), and skim milk (orange bars). The same letters indicate no significant differences (*p* < 0.05). Different letters indicate significant differences (*p* < 0.05), with the levels ranked as a > b > c > d. Error bars represent the standard deviation of three independent experiments.

To determine the dominance of *W. cibaria* VR81 and *L. plantarum* R12 in mixed cultures during the fermentation of quinoa flour and various plant-based milk, an enumeration on MRS + 2% sucrose was conducted. The results showed variations across the fermented matrices (Figure 9). In the fermented quinoa, *L. plantarum* exhibited the highest dominance with a concentration of 8.47 log CFU/g after 16 h of incubation, followed by soy milk with 8.14 log CFU/g, skim milk (7.79 log CFU/g), oat milk (7.78 log CFU/g) and coconut milk with the lowest concentration of 7.74 log CFU/g. 

The load of *W. cibaria* closely followed *L. plantarum*, with the highest concentration observed in the fermented quinoa (8.34 log CFU/g). *W. cibaria* displayed dominance in coconut milk with a concentration of 7.94 log CFU/g, while its lowest concentration in skim milk was at 7.59 log CFU/g.

## 4. Discussion

The properties of fermented dairy products derived from raw camel and goat milk can also be attributed to their natural microbiota, predominantly composed of lactic acid bacteria (LAB). These LAB strains possess diverse characteristics that offer considerable potential for application in the food industry [58,59] especially in the case of dairy [16,60]. Numerous studies have identified *Enterococcus* as the predominant lactic acid bacteria in camel milk, followed by *Leuconostoc*, *Lactococcus*, and lactobacilli, while *Weissella* species are less commonly found in this matrix [61,62,63]. In goat milk, the *Lactobacillaceae* family was the most dominant, comprising a significant majority of the bacterial community. This was followed by *Streptococcaceae* and *Enterobacteriaceae*, while the *Weissella* genus was the least prevalent [16,64]. The antimicrobial properties of LAB are crucial, as they support the ecological competition that allows these bacteria to establish dominance. This ability lets LAB drive the desired bio-based modifications, thereby enhancing the overall quality of food products [59]. For example, LAB contribute to the production of essential metabolites, such as organic acids, bacteriocins, reuterin, diacetyl, reutericyclin, acetoin, and hydrogen peroxide. These metabolites serve as biopreservative agents, inhibiting the growth of harmful microorganisms and ensuring the safety and extended shelf life of various fermented foods [59]. Many studies have reported the antimicrobial potential of *L. plantarum* and *W. cibaria* in inhibiting various foodborne pathogens, including *E. coli*, *L. monocytogenes*, and *S. aureus*. These LAB strains have also been effective against certain fungi, such as *Penicillium expansum*, *Aspergillus niger*, and *Botrytis cinerea* [42,59,65], suggesting their suitability as natural antimicrobial alternatives [66,67,68]. The growing interest in plant-based alternatives to cheeses and yogurts has led to the exploration of lactic acid bacteria (LAB) in the non-dairy sector. Numerous studies have demonstrated the efficacy of LAB in fermenting plant-based products, with soy being one of the most commonly used dairy alternatives [20]. Recently, several studies have started exploring the potential of quinoa for the formulation of non-dairy beverages, highlighting its status as an excellent source of protein, fiber, and antioxidants. However, these studies have primarily focused on the chemical and rheological properties of quinoa without addressing the impact of LAB as starters in quinoa fermentation, particularly regarding improvements in biocontrol and bio-fortification of the resulting quinoa beverages.

This study aimed to isolate and characterize indigenous LAB from raw Algerian dairy products with the ability to produce antimicrobial compounds to perform a wide polyphasic characterization. Given that limited studies have explored the application of LAB strains isolated from dairy sources in the fermentation process of plant-based products, our research further investigated the potential application of dairy LAB, both in single and co-culture, as a probiotic starter for fermenting plant-based products, using quinoa flour as a food matrix model. The experimental approach adopted in this work highlighted several novel aspects in relation to the existing scientific literature in the field.

The phenotypical and biochemical identification of LAB strains isolated from raw and fermented camel and goat milks showed the highest presence of *Enterococcus* sp. in all of the explored food niches. Many studies have reported a higher incidence of *Enterococcus* sp. in camel and goat dairy products, which is directly associated with raw materials, milking practices and the surroundings of animal sheds or farms [62,69,70]. A recent review papers underline the dual role of the *Enterococcus* genus in the food industry: on the one hand, *Enterococcus* species are recognized for their beneficial applications in food fermentation and probiotic properties; on the other hand, certain strains raise safety concerns due to their association with antibiotic resistance and virulence genes, underscoring the need for rigorous safety evaluations when considering these bacteria for use in food products [69,71]. Considering non-*Enterococcus* candidate LAB isolates, the best-performing biotypes were selected based on an evaluation of their antimicrobial potential against a large spectrum of foodborne pathogens, including *L. innocua*, *P. aeruginosa*, *E. coli*, *S. enterica*, and *S. aureus*. We followed this criterion, recognizing that antimicrobial activity is a critical property for both pro-technological and probiotic characteristics. The selection of an effective starter or probiotic culture involves enhancing the hygienic quality of the final product by inhibiting the proliferation of pathogenic and spoilage microorganisms [72,73]. Notably, the selected strains, in particular those from camel milk, displayed remarkable inhibition zones/antagonism against *L. innocua*, *P. aeruginosa*, and *S. aureus*. This strong antagonistic effect is primarily attributed to the production of various antimicrobial metabolites, including organic acids, bacteriocins, and hydrogen peroxide. Among these, organic acids are often considered the primary contributors to LAB’s inhibitory action, as their accumulation lowers the pH of the surrounding environment, thereby creating unfavorable conditions for the proliferation of these pathogens [42,74]. This underscores camel and goat milk’s potential as a reservoir of LAB with antimicrobial features of interest in designing bio-based food applications [16,63]. Moreover, numerous studies have extensively documented camel milk’s in vitro efficacy against both Gram-positive and Gram-negative bacteria [16,75,76]. The molecular characterization revealed that the 12 LAB strains exhibiting the highest inhibitory potential were *W. cibaria* (58.3%) and *L. plantarum* (41.7%). The *Lactiplantibacillus* community isolated from different mammal milks demonstrated wider diversity. In a recent study, the *Lactiplantibacillus* association from camel milk, with 4 out of 16 strains identified as *L. plantarum*, accounted for 25% of the consortium’s composition [77]. Additionally, Mercha et al. identified that among 18 isolates from Moroccan camel milk, only 4 strains exhibited homology with *W. cibaria* [78]. Merzouk et al. previously noted that lactococci emerge as the dominant LAB in Algerian camel milk, whereas lactobacilli occur in lower concentrations [79]. Additionally, Bakr-shori (2017) reported that some *Weissella* strains, including *Weissella confusa*, were isolated from fermented camel milk with a relatively low frequency [13]. Given the limited presence and documented reports of these bacteria in Algerian camel and goat milk, our study specifically concentrates on the investigation of these potential LAB strains isolated from spontaneously fermented camel milk. The antimicrobial potential of the cell-free supernatant of the selected LAB isolates has been tested against *L. innocua* ATCC 33090 and *P. aeruginosa* ATCC 27853. The results revealed that the inhibitory ability of *W. cibaria* strains VR81, LVT1, and *L. plantarum* strain R12 against *L. innocua* and *P. aeruginosa* was not only due to organic acid production but also indicate the presence of a substance with a proteinaceous nature that is sensitive to a proteolytic enzyme and stable at high temperatures (60–90 °C) and acidic conditions (pH 3 to 6.8). Peng et al. obtained similar results when they subjected plantaricin LP 21-2 produced by *L. plantarum* to high temperatures and different ranges of pH: 2–5 [80]. Furthermore, a study conducted by Maślak et al. on the antagonistic potential of *W. cibaria* against *L. innocua* and *P. aeruginosa* confirms our findings since *W. cibaria* completely inhibited the growth of the two target pathogens [4]. Arrioja-Bretón et al. found also that the anti-Listerial activity in neutralized CFS of *L. plantarum* is associated with peptides or proteins [81]. The observed loss of inhibitory activity after treatment with trypsin and chymotrypsin further supports this hypothesis.

Extensive literature, including studies conducted by Zhu et al. and Reale et al. [82,83], highlights the significant salt tolerance and resilience to low pH exhibited by *W. cibaria*, *Lacticaseibacillus casei*, *Lacticaseibacillus paracasei* subsp. *paracasei*, and *Lacticaseibacillus rhamnosus*. This distinctive trait is widely recognized as a key factor in the selection of promising probiotics. Based on this, we investigated the acid and bile salt tolerance of our strains to assess their potential as probiotic candidates. The results showed that the selected bioactive strains have good resistance to low pH, with the *L. plantarum* R12 and *W. cibaria* strain VR81 exhibiting the highest viability at both pH 2 and pH 3. In contrast to prior studies [84,85], our *W. cibaria* strains exhibited remarkable resistance even under challenging acidic conditions (pH 2.0), maintaining viability after 4 h incubation, thus demonstrating a robust adaptability to acidic environments. In parallel, the bile salt tolerance results demonstrated robust resistance in the presence of 2% oxgall, with even increased growth in 0,5% oxgall observed in both *W. cibaria* strains VR81, LVT1 and *L. plantarum* strain R12. Similar results were obtained in previous studies focusing on *W. cibaria* [85] and *L. plantarum* [86]. The exposure of bioactive LAB strains to 3 mg/mL of pepsin for 4 h demonstrated notable tolerance, although a slight decrease in growth was observed. This aligns with previous studies conducted by Quattrini et al. and Selmi et al. that also reported decreased growth and notable viability reduction in *W. cibaria* and *L. plantarum* when exposed to 3 mg/mL pepsin at pH 2.5 [42,87].

The ability of a probiotic strain to auto-aggregate is crucial in determining its capacity to adhere to various parts of the body, including the mucosal surfaces of oral, gastrointestinal, and urogenital tracts [88]. The tests indicated a substantial increase in auto-aggregation after 24 h for both *W. cibaria* (VR81 and LVT1) and *L. plantarum* R12. Previous studies have highlighted the significant role of longer incubation periods in enhancing auto-aggregation. Lakra et al. reported auto-aggregation percentages in *Laciplantibacillus* and *Weissella* species ranging from 18% to 79% after a 4 h period [84]. In the co-aggregation assay with *L. innocua*, both LAB strains exhibited consistent performance, with *W. cibaria* VR81 demonstrating the highest adherence percentages, followed closely by *L. plantarum* R12. These findings are consistent with similar results reported by Selmi et al. and Lakra et al. [42,84].

Adhesion to the host is a key criterion for selecting potential probiotic bacteria. By attaching to the intestinal mucosa, probiotics can protect against enteropathogens by competing for host–cell binding sites. Additionally, this adhesion enhances the interaction with the host, leading to temporary colonization and increased transit time in the gut, which allows probiotics to exert their beneficial effects more effectively [89]. The results of the adhesion ability to Caco-2 cells of the two bioactive strains indicate that *W. cibaria* VR81 and LVT1 exhibited higher adhesion capacity than the reference probiotic *L. plantarum* 299, which was used as a control. This enhanced adhesion can be related to the ability of lactic acid bacteria to produce both homopolysaccharides and heteropolysaccharides. These polysaccharides, which can either stay attached to the bacterial cells or be released into their environment, significantly influence bacterial aggregation, biofilm formation, and surface adhesion [89,90]. Consequently, the surface properties of bacteria and the type and amount of EPS they produce are crucial for their colonization and survival in a host [91]. Our findings are consistent with those reported by Huang et al., which highlight the superior adhesion ability of *W. cibaria* strains compared to *L. rhamnosus* GG [92]. This underscores the strong adhesive potential of *W. cibaria*, a crucial factor in considering its probiotic applications.

The metabolic activities of LAB, such as the synthesis of acetic acid, ethanol, aromatic compounds, and exopolysaccharides, are of great technological interest. Such capacities underline their broad application in the fermentation of various food matrices, where they contribute distinctive flavors, textures, and biocontrol attributes. The proteolytic activity of LAB enables them to break down peptides and proteins, leading to the generation of a variety of metabolites that enhance the flavor and texture of various food products [93]. The proteolytic activity assays revealed that all selected LAB strains effectively degraded casein in skim milk across concentrations of 3%, 5%, and 10%, with *W. cibaria* strains VR81 and *L. plantarum* R15 exhibiting the highest levels of activity. On the other hand, *L. plantarum* strain R12 was the only strain exhibiting low lipolytic activity. In earlier studies, Silva et al. found that traditional Algerian butter is a relevant source of LAB strains with lipolytic activity [94]. Conversely, LAB isolates with lipolytic abilities were missing in fermented milk and cheese, thus emphasizing the variability of this enzymatic profile across different dairy products. Analysis of the organic acids produced by the isolated LAB grown in MRS broth for 24 h revealed that acetic acid was the most abundant, surpassing 36 g/L for both *W. cibaria* (VR81 and LVT1) and *L. plantarum* R12. Additionally, *L. plantarum* was found to produce a high concentration of fumaric acid. In contrast to our findings, previous analogous studies conducted by Lim et al. on *W. cibaria* [95] and Selmi et al. on *L. plantarum* [42], reported that lactic acid was the most abundant (6.46 mg/mL; 13 g/L) followed by acetic acid and citric acid (2.78 mg/mL 0.88 mg/mL respectively). Similar to our findings, Selmi et al. noted that only one strain of *L. plantarum* could produce fumaric acid with a concentration of 6.71 mg/L.

Regarding EPS, all *W. cibaria* isolates exhibited a strong production when sucrose was used as the main sugar source. We observed the highest EPS level in *W. cibaria* VR81 (4.7 mg/mL), while the lowest amount was produced by *L. plantarum R15* strain (1.08 mg/mL) after 24 h of incubation. These findings are consistent with those reported by Quatrini et al. and Kumari et al., who noted high EPS production by *W. cibaria* cultivated in a solid sucrose-rich medium, while only minimal or not observable EPS amounts were produced by *Lacticaseibacillus rhamnosus* [83,85].

Vitamin-producing microorganisms, particularly LAB, offer a natural and economically sustainable option compared to fortifying food with chemically synthesized vitamins. Analysis of riboflavin levels in CDM medium revealed that mutant *W. cibaria* VR81d and *L. plantarum* R12d exhibited higher levels of B2 vitamin production, with the highest amount produced by the mutant *W. cibaria* VR81d. According to Iniaki et al., analysis of the rib operon leader region in *W. cibaria* cultures treated with roseoflavin using DNA sequencing revealed several mutations (G14T, G15T, T16G, C23T, A59C, G87A, G109A, A115G, and C120T), along with a deletion (ΔG15) at the FMN riboswitch. These genetic mutations may be contributing factors to the increased riboflavin production phenotype observed in the mutant strains [96]. Our results align with a study conducted by Hernández-Alcántara et al., which reported the capacity of mutant *W. cibaria* strains to overproduce riboflavin in RAM medium [97]. Another study conducted by Ge et al. identified *L. plantarum* as the highest riboflavin producer, with a content of 0.703 mg/L [98]. Notably, our findings show even higher riboflavin content in both *W. cibaria* VR81 and *L. plantarum* R12.

Our study aimed to develop a quinoa-based beverage using a co-culture of the most promising selected *W. cibaria* VR81 and *L. plantarum* R12 isolated from Algerian dairy products/camel milk. The two bioactive strains, VR81 and R12, demonstrated the effective acidification of the quinoa matrix, as evidenced by a decrease in pH from 6.07 to 3.98 after 5 h of incubation, with the presence of a distinct coagulum. This aligns with the findings of Väkeväinen et al., who noted comparable pH evolution during *L. plantarum* fermentation of quinoa flour [24]. The bioactive strains were also investigated for their biocontrol capacity in the fermentation of quinoa. In challenge tests against *L. innocua*, the latter was completely inhibited for up to 28 days in mixed culture and single culture using *L. plantarum* strain R12. LAB are known to produce diverse antimicrobial compounds that can hinder pathogenic bacteria growth. The combined action of *L. plantarum* R12 and *W. cibaria* VR81 in mixed culture may result in a synergistic effect, leading to the complete inhibition of *L. innocua*. On the other hand, in single-culture fermentation, at T0, the effectiveness of *L. plantarum* R12 against *L. innocua*, marked by complete inhibition, confirms its strong antimicrobial activity against this pathogen. In their study, Yin et al. affirmed the in vivo inhibitory potential of the co-culture of *L. plantarum* and *Pediococcus pentosaceus* species in the biocontrol of fresh cantaloupe stored at 4 °C against *Listeria monocytogenes* [99].

The effectiveness of the selected *L. plantarum* R12 and *W. cibaria* VR81 strains, in conducting a successful fermentation process, was supported by the high and stable CFU counts observed after 28 days in the final quinoa product. The production of organic acids and bioactive compounds by these LAB not only enhances the organoleptic properties of the final product but also serves as a natural bio preservative, inhibiting the growth of undesirable microorganisms. Moreover, the high LAB count suggests the potential of our fermented quinoa enriched with riboflavin to serve as a source of probiotics, offering potential health benefits to consumers.

In conclusion, this study explored the potential use of Algerian dairy *W. cibaria* and *L. plantarum* as probiotic starters and bioprotective cultures for developing an enriched quinoa-based beverage fortified with B2. Our findings indicate that these bioactive strains have desirable probiotic properties, including resilience to biotic and abiotic stressors, strong adhesion abilities, and high antagonistic activity, especially against *L. innocua*. Moreover, combining *W. cibaria* and *L. plantarum* in a co-culture resulted in faster acidification of the quinoa matrix, thereby accelerating the fermentation process and providing additional benefits compared to single-culture fermentation. *L. plantarum* was included for its strong antimicrobial activity and complete inhibition of *Listeria* growth during in vivo testing, while *W. cibaria* was chosen for its high EPS production, which significantly improved the texture and stability of the fermented quinoa beverage. These complementary traits demonstrate the broader potential of using co-cultures to optimize the fermentation process by enhancing both the safety and the functional properties of the final product. Overall, our study supports cross-over as a driver of innovation in the field of food fermentation. In fact, the strains of dairy origin are excellent candidates for fermenting plant-based products, enhancing safety and functional/nutritional properties, such as B2 vitamin fortification and biocontrol solutions. This study contributes to demonstrating the potential of microbial resources for the development of sustainable solutions in the agri-food sector [100].

## Figures and Tables

**Table 1:** Origin (source and geographical region) and antagonistic activity of LAB strains against *L. monocytogenes*, *S. aureus*, and *P. aeruginosa* using the agar disk diffusion assay. The diameter of inhibition zones is expressed in mm. Mean values and standard deviations of three replicate measurements are provided.

Strain	Species	Source	Geographical Region	Lm	Sa	Pa
15	*L. plantarum*	Camel leben	Bouktoub	20 ± 0.89	16 ± 1.54	17.33 ± 1.86
17	*L. plantarum*	Jben	Oran	20.33 ± 0.51	16.66 ± 0.51	21.5 ± 1.73
12	*L. plantarum*	Camel leben	Biskra	25 ± 0.00	10 ± 0.89	22.66 ± 0.51
OL2	*L. plantarum*	Jben	Msila	20.66 ± 1.86	9.66 ± 0.51	19.33 ± 1.86
ME7	*W. cibaria*	Raw goat milk	Saida	22.33 ± 1.36	8.66 ± 1.36	19.33 ± 1.36
ME10	*L. plantarum*	Raw goat milk	Tlemcen	20.33 ± 1.03	12 ± 1.78	23 ± 1.78
ME1	*W. cibaria*	Jben	Tlemcen	17 ± 1.78	11.33 ± 2.06	19 ± 2.36
ME9	*W. cibaria*	Raw goat milk	Saida	17 ± 1.54	8.33 ± 1.36	25 ± 0.00
ME5	*W. cibaria*	Goat leben	Oran	16.33 ± 1.36	12.5 ± 1.73	19.66 ± 1.36
ME101	*W. cibaria*	Camel leben	Bechar	17 ± 1.78	9.33 ± 1.03	19 ± 0.89
VR81	*W. cibaria*	Raw camel milk	Djelfa	24.33 ± 0.51	14.66 ± 1.36	22.66 ± 0.51
LVT1	*W. cibaria*	Raw camel milk	Oued Souf	19.33 ± 0.51	6.66 ± 1.03	16.66 ± 1.86
B7(Control)	*L. mensenteroides*	Raw camel milk	/	20 ± 0.31	10.03 ± 0.51	18 ± 0.31

Lm, *L. monocytogenes*; Sa, *Staphylococcus aureus*; Pa, *Pseudomonas aeruginosa*.

**Table 2:** Effect of temperature, pH, protease, and detergents on the antimicrobial activity of the CFS from the selected LAB strains against *L. innocua*. The diameter of the inhibition zones is expressed in mm. The mean values and standard deviations from three replicates are presented.

Strains				
		12	VR81	LVT1
Temperature	60 °C/30 min	19.1 ± 0.44	25 ± 0.35	23 ± 0.44
	70 °C/30 min	18.8 ± 0.22	23.8 ± 0.10	21.4 ± 0.44
	80 °C/30 min	20 ± 0.51	25.6 ± 0.25	22.5 ± 0.51
	90 °C/30 min	15 ± 0.44	18 ± 0.44	17.1 ± 0.36
	100 °C/15 min	0 ± 0.00	0 ± 0.00	0 ± 0.00
	120 °C/15 min	0 ± 0.00	0 ± 0.00	0 ± 0.00
pH	3	18.4 ± 0.25	19.2 ± 0.45	17.8 ± 0.68
	6	20 ± 0.68	25.4 ± 0.32	22.5 ± 0.50
	9	2 ± 0.00	0 ± 0.00	0 ± 0.00
Protease	Trypsin	0 ± 0.00	0 ± 0.00	0 ± 0.00
	chymostripsin	0 ± 0.00	0 ± 0.00	0 ± 0.00
Detergent	Tween80	22 ± 0.25	25.3 ± 0.24	22.9 ± 0.89
	Urea	9.5 ± 0.76	11 ± 0.51	10.5 ± 0.69

**Table 3:** Auto-aggregation results of *W. cibaria* and *L. plantarum* strains after 4 and 24 h of incubation with analysis performed in duplicate.

Strain	T4	T24
Wc VR81	37.8% ± 2.10%	65.7% ± 1.25%
Wc LVT1	21.0% ± 4.68%	48.0% ± 2.08%
Lp R12	27.0% ± 2.88%	55.6% ± 3.12%

Wc, *W. cibaria*; Lp, *L. plantarum*.

**Table 4:** Co-aggregation results of *W. cibaria* and *L. plantarum* strains with *L. innocua* after 4 h (T4) and 24 h (T24) of incubation with analysis performed in duplicate.

Strain	T4	T24
Wc VR81	21.0% ± 0.99%	21.1% ± 1.75%
Wc LVT1	16.5% ± 0.87%	15.1% ± 4.38%
Lp R12	19.1% ± 1.88%	18.6% ± 2.62%

Wc, *W. cibaria*; Lp, *L. plantarum*.

**Table 5:** Antibiotic susceptibility profiles of *W. cibaria* VR81, LVT1 and *L. plantarum* R12: results are expressed as resistant (R), susceptible (S), and intermediate (I).

Antibiotics/Strains	Wc VR81	Lp R12	Wc LVT1
Ampicillin (10 micrograms)	I	S	S
Gentamycin (10 micrograms)	S	S	S
Kanamycin (10 micrograms)	R	R	R
Streptomycin (10 micrograms)	S	R	S
Tetracycline (30 micrograms)	S	S	R
Chloramphenicol (30 micrograms)	S	S	S
Vancomycin (30 micrograms)	R	R	R
Clindamycin (2 micrograms)	S	S	S
Erythromycin (10 micrograms)	R	R	S

Wc, *W. cibaria*; Lp, *L. plantarum*.

**Table 6:** Quantification of organic acids in cell-free supernatants of LAB strains grown in MRS broth determined using HPLC. Results (mg L^−1^) are presented as the mean values and standard deviations from two measurements. The same letters indicate no significant differences (*p* < 0.05). Different letters indicate significant differences (*p* < 0.05), with the levels ranked as e > d > c > b > a.

Strain	Tartaric Acid	Malic Acid	Ascorbic Acid	Lactic Acid	Acetic Acid	Citric Acid	Succinic Acid	Fumaric Acid
LVT1	2121.44 ± 16.02 ^d^	2887.01 ± 190.49 ^cd^	453.88 ± 9.1 ^a^	6953.42 ± 94.24 ^ab^	34,388.18 ± 1764.18 ^b^	1213.79 ± 225.91 ^a^	2634.49 ± 282.34 ^b^	0 ± 0 ^a^
VR81	2398.44 ± 38.85 ^d^	1739.3 ± 237.73 ^b^	461.99 ± 34.46 ^ab^	5976.35 ± 200.77 ^a^	36,967.96 ± 1086.5 ^b^	2053.68 ± 160.99 ^c^	2514.58 ± 305.02 ^b^	0 ± 0 ^a^
OL2	882.77 ± 23.02 ^a^	0 ± 0 ^a^	646.59 ± 45.67 ^c^	10,412.42 ± 1853.48 ^c^	30,741.09 ± 1466.75 ^a^	972.61 ± 306.62 ^a^	0 ± 0 ^a^	0 ± 0 ^a^
R12	1229.96 ± 25.15 ^a^	2349.92 ± 122.14 ^bc^	746.33 ± 0.17 ^e^	10,033.48 ± 184.36 ^c^	34,641.98 ± 697.99 ^b^	1282.6 ± 225.84 ^a^	9735.66 ± 434.46 ^c^	13.35 ± 1.45 ^d^
R15	1596.71 ± 2.43 ^b^	2395.5 ± 313.83 ^bc^	729 ± 12.77 ^e^	8952.03 ± 557.79 ^ab^	36,402.22 ± 1538.56 ^b^	1111.94 ± 59.49 ^a^	9741.39 ± 133.42 ^c^	8.95 ± 0.26 ^c^
17	1579.41 ± 2.88 ^b^	1888.59 ± 52.07 ^bc^	744.1 ± 3.33 ^e^	9441.7 ± 226.7 ^c^	32,969.17 ± 515.6 ^ab^	1466.06 ± 105.46 ^b^	10,666.67 ± 353.23 ^cd^	8.55 ± 1.53 ^c^
ME1	1277.88 ± 14.15 ^ab^	2062.68 ± 79.47 ^bc^	655.67 ± 1.81 ^d^	5843.13 ± 61.7 ^a^	44,107.55 ± 126.41 ^c^	1030.82 ± 71.94 ^a^	0 ± 0 ^a^	2.26 ± 0.04 ^b^
ME7′	1161.62 ± 392.76 ^a^	2672.15 ± 726.44 ^bc^	544.25 ± 2.23 ^b^	4647.77 ± 538.55 ^a^	40,674.01 ± 5213.86 ^c^	881.67 ± 111.91 ^a^	0 ± 0 ^a^	0 ± 0 ^a^
ME10	1903.2 ± 58.11 ^bc^	3549.62 ± 124.86 ^d^	564.75 ± 44.86 ^bc^	10,468.55 ± 512.93 ^c^	31,300.1 ± 170.8 ^a^	1079.08 ± 66.92 ^a^	2157.52 ± 36.21 ^b^	0 ± 0 ^a^
ME9	2053.01 ± 71.04 ^d^	2315.22 ± 217.13 ^bc^	431.7 ± 2.84 ^a^	11,755.76 ± 46.28 ^c^	32,516.91 ± 774.84 ^a^	3430.48 ± 7.21 ^d^	0 ± 0 ^a^	0 ± 0 ^a^
ME5	1582.72 ± 21.52 ^b^	3074.67 ± 440.1 ^d^	376.88 ± 5.9 ^a^	10,701.5 ± 30.73 ^c^	26,154.67 ± 138.45 ^a^	4117.91 ± 19.37 ^e^	11,242.26 ± 132.54 ^d^	0 ± 0 ^a^
ME10′	2035.51 ± 14.83 ^cd^	1888.11 ± 280.49 ^bc^	631.91 ± 29.36 ^c^	9994.69 ± 405.58 ^c^	36,752.29 ± 1020.31 ^b^	1083.88 ± 51.78 ^a^	0 ± 0 ^a^	0 ± 0 ^a^

**Table 7:** Growth response (OD measurements) of LAB strains under various stress conditions. The assay was performed in duplicate.

Stress Condition	VR81	R12
C+: Osmotic stress (0 M sucrose)	2.09 ± 0.047	2.008 ± 0.056
Osmotic stress (0.2 M sucrose)	1.979 ± 0.083	0.963 ± 0.022
Osmotic stress (0.6 M sucrose)	1.0775 ± 0.022	0.14 ± 0.031
Osmotic stress (1 M sucrose)	0.589 ± 0.050	0.00 ± 0.00
Oxidative stress (62.5 µM H_2_O_2_)	0.07 ± 0.003	0.11 ± 0.0011
Oxidative stress (125 µM H_2_O_2_)	0.068 ± 0.0005	0.094 ± 0.006
Oxidative stress (220 µM H_2_O_2_)	0.074 ± 0.0011	0.09 ± 0.0016
pH 3	1.785 ± 0.012	1.341 ± 0.061
pH 7	2.152 ± 0.009	2.103 ± 0.016
pH 9	2.421 ± 0.004	2.233 ± 0.017
Growth at 65 °C	0.202 ± 0.018	0.298 ± 0.010
Growth at 4 °C	0.105 ± 0.059	0.125 ± 0.091
C+: Growth at 37 °C	2.308 ± 0.066	2.445 ± 0.032

## Data Availability

The datasets generated for this study are available upon request to the corresponding author.

## Supplementary Materials

The following supporting information can be downloaded at: https://www.mdpi.com/article/10.3390/microorganisms12102042/s1, Figure S1: pH variation of skim and plant-based milk using single cultures of *W. cibaria* and *L. plantarum*; Table S1: morphological, physiological and biochemical characteristics of the isolated LAB strains from different Algerian dairy products; Table S2: Fermentation profile of *W. cibaria* and *L. plantarum* selected strains using API 50 CHL; Table S3: Proteolytic, amylolytic and lipolytic activity of the selected *W. cibaria* and *L. plantarum* strains; Table S4: Exopolysaccharide quantification of the selected LAB strains using sucrose as a sugar source.
