# Supplementary material for: Cross-Over Application of Algerian Dairy Lactic Acid Bacteria for the Design of Plant-Based Products: Characterization of Weissella cibaria and Lactiplantibacillus plantarum for the Formulation of Quinoa-Based Beverage
Source: Microorganisms. 2024 Oct 9;12(10):2042. doi: 10.3390/microorganisms12102042 (PMC11510054; doi:10.3390/microorganisms12102042)
Supplement: Supplementary file 1 [file microorganisms-12-02042-s001.zip › microorganisms-3220362-supplementary.pdf]

Table S1. Morphological, physiological and biochemical characteristics of the isolated LAB strains from different Algerian dairy products.

Strain	Morphological characteristics	Biochemical characteristics			Physiological characteristics							
		CO2	BEA	ADH	4°C	15°C	37°C	45°C	pH 4,8	pH 6,8	3% NaCl	6,8% NaCl
**C2**	Gram+, cocci	+	-	-	-	+	+	+	+	+	+	+
**G**	Gram+, cocci	+	-	-	-	+	+	+	+	+	+	+
**ME1**	Gram+, cocci	+	-	-	-	+	+	+	+	+	+	+
**ME2**	Gram+, cocci	+	-	-	-	+	+	+	+	+	+	+
**ME3**	Gram+, cocci	+	-	-	-	+	+	+	+	+	+	+
**ME4**	Gram+, cocci	+	-	-	-	+	+	+	+	+	+	+
**ME5**	Gram+, cocci	+	-	-	-	+	+	+	+	+	+	+
**ME6**	Gram+, cocci	-	+	+	+	+	+	+	+	+	+	+
**ME7**	Gram+, cocci	+	+	+	+	+	+	+	+	+	+	+
**ME8**	Gram+, cocci	-	+	+	+	+	+	+	+	+	+	+
**ME9**	Gram+, cocci	+	+	+	+	+	+	+	+	+	+	+
**ME10**	Gram+, cocci	+	-	+	+	+	+	+	+	+	+	+
**MS1**	Gram+, cocci	+	+	-	+	+	+	+	+	+	+	+
**MS2**	Gram+, cocci	+	+	-	+	+	+	+	+	+	+	+
**MS3**	Gram+, cocci	+	+	-	+	+	+	+	+	+	+	+
**MS4**	Gram+, cocci	-	+	+	+	+	+	+	+	+	+	+
**MS5**	Gram+, cocci	-	+	+	+	+	+	+	+	+	+	+
**MS6**	Gram+, cocci	-	+	+	+	+	+	+	+	+	+	+
**MS7**	Gram+, cocci	+	+	-	+	+	+	+	+	+	+	+
**MS8**	Gram+, cocci	-	+	+	+	+	+	+	+	+	+	+
**MS9**	Gram+, cocci	+	+	-	+	+	+	+	+	+	+	+
**MS10**	Gram+, cocci	+	+	-	+	+	+	+	+	+	+	+
**VRa1**	Gram+, cocci	-	+	+	+	+	+	+	+	+	+	+
**VRa2**	Gram+, cocci	-	+	+	+	+	+	+	+	+	+	+
**VRa3**	Gram+, cocci	-	+	+	+	+	+	+	+	+	+	+
**VRa4**	Gram+, cocci	-	+	+	+	+	+	+	+	+	+	+
**VRa5**	Gram+, cocci	-	+	+	+	+	+	+	+	+	+	+
**VRa6**	Gram+, cocci	-	+	+	+	+	+	+	+	+	+	+
**VR81**	Gram+, Irregular rod	+	+	+	+	+	+	+	+	+	+	+
**LVT1**	Gram+, Irregular rod	+	+	+	+	+	+	+	+	+	+	+
**LVT1′**	Gram+, cocci	+	+	+	+	+	+	+	+	+	+	+

**LVT2**	Gram+, Irregular rod	+	+	+	+	+	+	+	+	+	+	+
**LVT3**	Gram+, cocci	-	+	+	+	+	+	+	+	+	+	+
**LVT4**	Gram+, cocci	-	+	+	+	+	+	+	+	+	+	+
**LVT5**	Gram+, cocci	-	+	+	+	+	+	+	+	+	+	+
**LVT6**	Gram+, cocci	-	+	+	-	+	+	+	+	+	+	+
**LVT7**	Gram+, Irregular rod	+	+	+	+	+	+	+	+	+	+	+
**LVT8**	Gram+, Irregular rod	+	+	+	+	+	+	+	+	+	+	+
**LVT9**	Gram+, cocci	-	+	+	+	+	+	+	+	+	+	+
**LVT10**	Gram+, cocci	-	+	-	+	+	+	+	+	+	+	+
**LVT11**	Gram+, cocci	-	+	-	-	+	+	+	+	+	+	+
**LVT12**	Gram+, cocci	-	+	+	+	+	+	+	+	+	+	+
**LVT13**	Gram+, cocci	-	+	+	+	+	+	+	+	+	+	+
**LVT14**	Gram+, cocci	-	+	+	+	+	+	+	+	+	+	+
**LVT14′**	Gram+, cocci	-	+	+	+	+	+	+	+	+	+	+
**LVT15**	Gram+, cocci	-	+	+	+	+	+	+	+	+	+	+
**CaT1**	Gram+, cocci	-	+	+	+	+	+	+	+	+	+	+
**CaT2**	Gram+, cocci	-	+	+	+	+	+	+	+	+	+	+
**CaT3**	Gram+, cocci	-	+	+	+	+	+	+	+	+	+	+
**CaT4**	Gram+, cocci	-	+	+	+	+	+	+	+	+	+	+
**CaT5**	Gram+, cocci	-	+	+	+	+	+	+	+	+	+	+
**CaT6**	Gram+, cocci	-	+	+	+	+	+	+	+	+	+	+
**CaT7**	Gram+, cocci	-	+	+	+	+	+	+	+	+	+	+
**CaT8**	Gram+, cocci	-	+	-	+	+	+	+	+	+	+	+
**CaT9**	Gram+, cocci	-	+	+	+	+	+	+	+	+	+	+
**CaT10**	Gram+, cocci	-	+	+	+	+	+	+	+	+	+	+
**CaT11**	Gram+, cocci	-	+	+	+	+	+	+	+	+	+	+
**CaT12**	Gram+, cocci	-	+	-	+	+	+	+	+	+	+	+
**CaT13**	Gram+, cocci	-	+	+	+	+	+	+	+	+	+	+
**CaT14**	Gram+, cocci	-	+	+	+	+	+	+	+	+	+	+
**CaT15**	Gram+, cocci	-	+	+	+	+	+	+	+	+	+	+
**CaT16**	Gram+, cocci	-	+	+	-	+	+	+	+	+	+	+
**CaT17**	Gram+, cocci	-	+	+	+	+	+	+	+	+	+	+
**CaT18**	Gram+, cocci	-	+	+	+	+	+	+	+	+	+	+
**VR1**	Gram+, cocci	+	-	-	+	+	+	+	+	+	+	+
**VR2**	Gram+, cocci	+	-	-	+	+	+	+	+	+	+	+

**VR3**	Gram+, cocci	+	-	-	+	+	+	+	+	+	+	+
**VR4**	Gram+, cocci	+	-	-	+	+	+	+	+	+	+	+
**VR5**	Gram+, cocci	+	-	-	+	+	+	+	+	+	+	+
**VR6**	Gram+, cocci	+	-	-	+	+	+	+	+	+	+	+
**VR8**	Gram+, cocci	+	-	-	+	+	+	+	+	+	+	+
**MRa1**	Gram+, cocci	+	-	-	+	+	+	+	+	+	+	+
**MRa2**	Gram+, cocci	+	-	+	+	+	+	+	+	+	+	+
**MRa3**	Gram+, cocci	+	-	-	+	+	+	+	+	+	+	+
**MRa4**	Gram+, cocci	+	-	-	+	+	+	+	+	+	+	+
**MRa5**	Gram+, cocci	+	-	-	+	+	+	+	+	+	+	+
**MRa6**	Gram+, cocci	+	-	-	+	+	+	+	+	+	+	+
**MRa7**	Gram+, cocci	+	-	-	+	+	+	+	+	+	+	+
**MRa8**	Gram+, cocci	+	-	-	+	+	+	+	+	+	+	+
**MRa9**	Gram+, cocci	+	-	-	+	+	+	+	+	+	+	+
**MRa10**	Gram+, cocci	+	-	+	+	+	+	+	+	+	+	+
**MRa11**	Gram+, cocci	+	-	-	+	+	+	+	+	+	+	+
**MRa12**	Gram+, cocci	+	-	-	+	+	+	+	+	+	+	+
**MRa13**	Gram+, cocci	+	-	-	+	+	+	+	+	+	+	+
**MRa14**	Gram+, cocci	+	-	-	+	+	+	+	+	+	+	+
**MRa15**	Gram+, cocci	+	-	-	+	+	+	+	+	+	+	+
**RS1**	Gram+, cocci	-	+	+	+	+	+	+	+	+	+	+
**RS2**	Gram+, cocci	-	+	+	+	+	+	+	+	+	+	+
**RS3**	Gram+, cocci	-	+	+	+	+	+	+	+	+	+	+
**RS4**	Gram+, cocci	-	+	+	+	+	+	+	+	+	+	+
**RS5**	Gram+, cocci	-	+	+	+	+	+	+	+	+	+	+
**RS6**	Gram+, cocci	-	+	+	+	+	+	+	+	+	+	+
**RS7**	Gram+, cocci	-	+	+	+	+	+	+	+	+	+	+
**RS8**	Gram+, cocci	-	+	+	+	+	+	+	+	+	+	+
**RS9**	Gram+, cocci	-	+	+	+	+	+	+	+	+	+	+
**M1**	Gram+, cocci	+	-	+	+	+	+	+	+	+	+	+
**M2**	Gram+, cocci	+	-	+	+	+	+	+	+	+	+	+
**M3**	Gram+, cocci	+	-	-	+	+	+	+	+	+	+	+
**M4**	Gram+, cocci	+	-	+	-	+	+	+	+	+	+	+
**M5**	Gram+, cocci	+	-	-	-	+	+	+	+	+	+	+
**M6**	Gram+, cocci	+	-	-	-	+	+	+	+	+	+	+

**M7**	Gram+, cocci	+	-	+	+	+	+	+	+	+	+	+
**M8**	Gram+, cocci	+	-	+	+	+	+	+	+	+	+	+
**M9**	Gram+, cocci	+	-	+	+	+	+	+	+	+	+	+
**M10**	Gram+, cocci	+	-	+	+	+	+	+	+	+	+	+
**CaB1**	Gram+, cocci	-	+	+	-	+	+	+	+	+	+	-
**CaB2**	Gram+, cocci	-	+	+	-	+	+	+	+	+	+	-
**CaB3**	Gram+, cocci	-	+	+	-	+	+	+	+	+	+	-
**CaB4**	Gram+, cocci	-	+	+	+	+	+	+	+	+	+	+
**CaB5**	Gram+, cocci	-	+	+	+	+	+	+	+	+	+	+
**CaB6**	Gram+, cocci	-	+	+	+	+	+	+	+	+	+	+
**CaB7**	Gram+, cocci	-	+	+	-	+	+	+	+	+	+	+
**CaB8**	Gram+, cocci	-	+	+	+	+	+	+	+	+	+	+
**CaB9**	Gram+, cocci	-	+	+	+	+	+	+	+	+	+	+
**CaB10**	Gram+, cocci	-	+	+	+	+	+	+	+	+	+	+
**CaB11**	Gram+, cocci	-	+	+	+	+	+	+	+	+	+	+
**CaB12**	Gram+, cocci	-	+	+	+	+	+	+	+	+	+	+
**CaB13**	Gram+, cocci	-	+	+	+	+	+	+	+	+	+	+
**CaB14**	Gram+, cocci	-	+	+	+	+	+	+	+	+	+	+
**CaB15**	Gram+, cocci	-	+	+	+	+	+	+	+	+	+	+
**CaB16**	Gram+, cocci	-	+	+	+	+	+	+	+	+	+	+
**LB1**	Gram+, rod	-	+	+	+	+	+	+	+	+	+	+
**LB2**	Gram+, rod	-	+	+	+	+	+	+	+	+	+	+
**LB3**	Gram+, rod	-	+	+	+	+	+	+	+	+	+	+
**LB4**	Gram+, rod	-	+	+	+	+	+	+	+	+	+	+
**LB5**	Gram+, rod	-	+	+	-	+	+	+	+	+	+	+
**LB6**	Gram+, rod	-	+	+	+	+	+	+	+	+	+	+
**LB7**	Gram+, rod	-	+	+	+	+	+	+	+	+	+	+
**LB8**	Gram+, rod	-	+	+	+	+	+	+	+	+	+	+
**LB9**	Gram+, rod	-	-	+	+	+	+	+	+	+	+	+
**LB10**	Gram+, rod	-	+	+	+	+	+	+	+	+	+	+
**LB11**	Gram+, rod	-	+	+	+	+	+	+	+	+	+	+
**LB12**	Gram+, rod	-	-	+	+	+	+	+	+	+	+	+
**LB13**	Gram+, rod	-	-	+	+	+	+	+	+	+	+	+
**LB14**	Gram+, rod	-	-	+	+	+	+	+	+	+	+	+
**LB15**	Gram+, rod	-	+	+	+	+	+	+	+	+	+	+

**LB16**	Gram+, rod	-	+	+	+	+	+	+	+	+	+	+
**LB17**	Gram+, rod	-	+	+	+	+	+	+	+	+	+	+
**LB18**	Gram+, rod	-	+	+	+	+	+	+	+	+	+	+
**LB19**	Gram+, rod	-	+	+	+	+	+	+	+	+	+	+
**LB20**	Gram+, rod	-	+	+	+	+	+	+	+	+	+	+
**VS1**	Gram+, cocci	-	+	+	-	+	+	+	+	+	+	+
**VS2**	Gram+, cocci	-	+	+	-	+	+	+	+	+	+	+
**VS3**	Gram+, cocci	-	+	+	+	+	+	+	+	+	+	+
**VS4**	Gram+, cocci	-	+	+	+	+	+	+	+	+	+	+
**VS5**	Gram+, cocci	-	+	-	-	+	+	+	+	+	+	+
**VS6**	Gram+, cocci	-	+	+	-	+	+	+	+	+	+	+
**VS7**	Gram+, cocci	-	+	+	+	+	+	+	+	+	+	+
**VS8**	Gram+, cocci	-	+	-	+	+	+	+	+	+	+	+
**VS9**	Gram+, cocci	-	+	-	+	+	+	+	+	+	+	+
**VS10**	Gram+, cocci	-	+	-	+	+	+	+	+	+	+	+
**CH1**	Gram+, cocci	-	+	+	+	+	+	+	+	+	+	+
**CH2**	Gram+, cocci	-	+	+	+	+	+	+	+	+	+	+
**CH3**	Gram+, cocci	-	+	+	-	+	+	+	+	+	+	+
**CH4**	Gram+, cocci	+	-	-	+	+	+	+	+	+	+	+
**CH5**	Gram+, cocci	+	-	+	+	+	+	+	+	+	+	+
**CH6**	Gram+, cocci	+	-	+	+	+	+	+	+	+	+	-
**CH7**	Gram+, cocci	-	+	+	+	+	+	+	+	+	+	+
**LB1a**	Gram+, rod	-	+	+	-	+	+	+	+	+	+	+
**LB2a**	Gram+, rod	-	+	+	-	+	+	+	+	+	+	+
**LB3a**	Gram+, rod	-	+	+	-	+	+	+	+	+	+	+
**LB4a**	Gram+, rod	-	+	+	-	+	+	+	+	+	+	+
**LB5a**	Gram+, rod	-	+	+	+	+	+	+	+	+	+	+
**LB1b**	Gram+, rod	-	+	+	+	+	+	+	+	+	+	+
**LB2b**	Gram+, rod	-	+	+	+	+	+	+	+	+	+	+
**LB3b**	Gram+, rod	+	-	-	+	+	+	+	+	+	+	+
**LB4b**	Gram+, rod	+	-	-	+	+	+	+	+	+	+	+
**LB5b**	Gram+, rod	+	-	-	+	+	+	+	+	+	+	+
**LB6b**	Gram+, rod	-	+	+	+	+	+	+	+	+	+	+
**M171**	Gram+, rod	-	+	+	-	+	+	+	+	+	+	+
**M172**	Gram+, rod	-	+	+	-	+	+	+	+	+	+	+

**M173**	Gram+, rod	-	+	+	-	+	+	+	+	+	+	-
**M174**	Gram+, rod	-	+	+	+	+	+	+	+	+	+	+
**M175**	Gram+, rod	-	+	+	+	+	+	+	+	+	+	+
**M176**	Gram+, rod	-	+	+	+	+	+	+	+	+	+	+
**M177**	Gram+, rod	-	+	+	+	+	+	+	+	+	+	+
**M178**	Gram+, rod	-	+	+	+	+	+	+	+	+	+	+
**MRV1**	Gram+, cocci	+	+	-	-	+	+	+	+	+	+	+
**MRV2**	Gram+, cocci	+	-	+	+	+	+	+	+	+	+	+
**MRV3**	Gram+, cocci	+	+	-	+	+	+	+	+	+	+	+
**MRV4**	Gram+, cocci	+	+	-	+	+	+	+	+	+	+	+
**MRV5**	Gram+, cocci	+	-	-	-	+	+	+	+	+	+	+
**MRV6**	Gram+, cocci	+	-	-	-	+	+	+	+	+	+	+
**MRV7**	Gram+, cocci	+	+	-	+	+	+	+	+	+	+	+
**MRV8**	Gram+, cocci	+	-	-	+	+	+	+	+	+	+	+
**MRV9**	Gram+, cocci	+	-	-	+	+	+	+	+	+	+	+
**MRV10**	Gram+, cocci	+	-	-	+	+	+	+	+	+	+	+
**VaC1**	Gram+, cocci	-	+	-	+	+	+	+	+	+	+	+
**VaC2**	Gram+, cocci	+	+	-	+	+	+	+	+	+	+	+
**VaC2′**	Gram+, cocci	+	+	-	+	+	+	+	+	+	+	+
**VaC3**	Gram+, cocci	-	+	-	+	+	+	+	+	+	+	+
**VaC4**	Gram+, cocci	-	+	-	+	+	+	+	+	+	+	+
**VaC5**	Gram+, cocci	-	+	+	+	+	+	+	+	+	+	+
**VaC6**	Gram+, cocci	-	+	+	+	+	+	+	+	+	+	+
**VaC7**	Gram+, cocci	-	+	+	-	+	+	+	+	+	+	+
**VaC8**	Gram+, cocci	+	+	+	-	+	+	+	+	+	+	+
**VaC8′**	Gram+, Irregular rod	+	+	+	+	+	+	+	+	+	+	+
**VaC9**	Gram+, cocci	-	+	-	+	+	+	+	+	+	+	+
**VaC10**	Gram+, cocci	+	+	+	+	+	+	+	+	+	+	+
**VaC11**	Gram+, Irregular rod	+	+	-	+	+	+	+	+	+	+	+
**VaC12**	Gram+, Irregular rod	+	+	+	+	+	+	+	+	+	+	+
**VaC13**	Gram+, cocci	-	+	+	+	+	+	+	+	+	+	+
**VaC14**	Gram+, cocci	-	+	+	+	+	+	+	+	+	+	+
**VaC15**	Gram+, cocci	-	+	+	+	+	+	+	+	+	+	+
**VaC16**	Gram+, cocci	-	+	+	+	+	+	+	+	+	+	+
**VaC17**	Gram+, Irregular rod	+	+	+	+	+	+	+	+	+	+	+

**VaC18**	Gram+, Irregular rod	+	+	+	+	+	+	+	+	+	+	+
**LS1**	Gram+, cocci	-	+	+	+	+	+	+	+	+	+	+
**LS2**	Gram+, cocci	-	+	+	-	+	+	+	+	+	+	+
**LS2′**	Gram+, cocci	-	+	+	-	+	+	+	+	+	+	+
**LS3**	Gram+, cocci	+	+	-	-	+	+	+	+	+	+	+
**LS4**	Gram+, cocci	+	+	-	-	+	+	+	+	+	+	+
**LS4′**	Gram+, cocci	-	+	-	+	+	+	+	+	+	+	+
**LS5**	Gram+, cocci	+	+	-	+	+	+	+	+	+	+	+
**LS6**	Gram+, cocci	-	+	+	+	+	+	+	+	+	+	+
**LS7**	Gram+, cocci	-	+	+	+	+	+	+	+	+	+	+
**LS7′**	Gram+, cocci	-	+	+	+	+	+	+	+	+	+	+
**LS8**	Gram+, cocci	-	+	+	+	+	+	+	+	+	+	+
**LS9**	Gram+, cocci	-	+	+	-	+	+	+	+	+	+	+
**LS10**	Gram+, cocci	+	+	+	-	+	+	+	+	+	+	+
**LS10′**	Gram+, cocci	-	+	+	+	+	+	+	+	+	+	+
**R1**	Gram+, rod	-	+	+	+	+	+	+	+	+	+	+
**R2**	Gram+, rod	-	+	+	+	+	+	+	+	+	+	+
**R3**	Gram+, rod	-	+	+	+	+	+	+	+	+	+	+
**R4**	Gram+, rod	+	+	+	+	+	+	+	+	+	+	+
**R5**	Gram+, rod	-	+	+	+	+	+	+	+	+	+	+
**R6**	Gram+, rod	-	+	+	-	+	+	+	+	+	+	+
**R7**	Gram+, rod	+	+	+	+	+	+	+	+	+	+	+
**R8**	Gram+, rod	-	+	+	+	+	+	+	+	+	+	+
**R9**	Gram+, rod	-	+	+	+	+	+	+	+	+	+	+
**R10**	Gram+, rod	-	+	+	+	+	+	+	+	+	+	+
**R11**	Gram+, rod	-	+	-	-	+	+	+	+	+	+	+
**R12**	Gram+, rod	-	+	+	+	+	+	+	+	+	+	+
**R13**	Gram+, rod	-	+	+	+	+	+	+	+	+	+	+
**R14**	Gram+, rod	-	+	+	+	+	+	+	+	+	+	+
**R16**	Gram+, rod	-	+	+	+	+	+	+	+	+	+	+
**R17**	Gram+, rod	+	+	+	+	+	+	+	+	+	+	+
**R18**	Gram+, rod	-	+	+	+	+	+	+	+	+	+	+
**R19**	Gram+, rod	-	+	+	+	+	+	+	+	+	+	+
**R20**	Gram+, rod	-	+	+	+	+	+	+	+	+	+	+
**S1**	Gram+, Irregular rod	+	+	+	+	+	+	+	+	+	+	+

**S2**	Gram+, Irregular rod	+	+	+	-	+	+	+	+	+	+	+
**S3**	Gram+, Irregular rod	+	+	+	+	+	+	+	+	+	+	+
**S4**	Gram+, cocci	+	+	+	+	+	+	+	+	+	+	+
**S5**	Gram+, cocci	+	-	+	+	+	+	+	+	+	+	+
**S6**	Gram+, cocci	+	+	+	+	+	+	+	+	+	+	+
**S7**	Gram+, cocci	+	+	+	-	+	+	+	+	+	+	+
**S8**	Gram+, Irregular rod	+	-	+	+	+	+	+	+	+	+	+
**S9**	Gram+, Irregular rod	+	+	+	+	+	+	+	+	+	+	+
**S10**	Gram+, Irregular rod	+	+	+	-	+	+	+	+	+	+	+
**S11**	Gram+, Irregular rod	+	+	+	-	+	+	+	+	+	+	+
**S12**	Gram+, Irregular rod	+	+	+	+	+	+	+	+	+	+	+
**S13**	Gram+, short rod	+	+	+	-	+	+	+	+	+	+	+
**S14**	Gram+, Irregular rod	+	-	+	-	+	+	+	+	+	+	+
**WS15**	Gram+, short rod	+	+	+	+	+	+	+	+	+	+	+

Table S2. Fermentation profile of *W. cibaria* and *L. plantarum* selected strains using API 50 CHL

Type of sugar	1	2	3	4	5	6	7	8	9	10	11	12	13	14	15	16	17	18	19	20	21	22	23	24	25
**Strain code**	Glycerol	Erythritol	D-arabinose	L-arabinose	Ribose	D-xylose	L-xylose	Adonitol	β methyl-D-xyloside	Galactose	Glucose	Fructose	Mannose	Sorbose	Rhamnose	Dulcitol	Inositol	Mannitol	Sorbotol	Methyl-D-mannoside	Methyl-D-glucoside	N-acetyl-glucosamine	Amygdalin	Arbutin	Esculin
**VR81**	-	-	-	+	+	-	-	-	-	+	+	+	+	-	-	-	-	+	+	-	-	+	-	+	+
**LVT1**	-	-	-	+	+	-	-	-	-	+	+	+	+	-	-	-	-	+	+	-	-	+	-	+	+
**R12**	-	-	-	-	-	-	-	-	-	-	+	+	-	-	-	-	-	-	-	-	-	-	-	-	-
**R15**	-	-	-	-	-	-	-	-	-	-	+	+	-	-	-	-	-	-	-	-	-	-	-	-	-
**R17**	-	-	-	-	-	-	-	-	-	-	+	+	-	-	-	-	-	-	-	-	-	-	-	-	-
**OL2**	-	-	-	-	-	-	-	-	-	+	+	+	-	-	-	-	-	-	-	-	-	-	-	-	-
**ME7**	-	-	-	+	+	-	-	-	-	+	+	+	+	-	-	-	-	+	+	-	-	+	-	+	+
**ME10**	-	-	-	+	+	-	-	-	-	+	+	+	+	-	-	-	-	+	+	-	-	+	-	+	+
**ME1**	-	-	-	+	+	-	-	-	-	+	+	+	+	-	-	-	-	+	+	-	-	+	-	+	+
**ME9**	-	-	-	+	+	-	-	-	-	+	+	+	+	-	-	-	-	-	+	-	-	+	-	+	+
**ME5**	-	-	-	+	+	-	-	-	-	+	+	+	+	-	-	-	-	+	+	-	-	+	-	+	+
**ME101**	-	-	-	+	+	-	-	-	-	+	+	+	+	-	-	-	-	+	+	-	-	+	-	+	+

**Type of sugar**	26	27	28	29	30	31	32	33	34	35	36	37	38	39	40	41	42	43	44	45	46	47	48	49
**Strain code**	Sal[illegible]	Ce[illegible]	M[illegible]	La[illegible]	M[illegible]	Su[illegible]	Tr[illegible]	In[illegible]	M[illegible]	Ra[illegible]	St[illegible]	Gl[illegible]	Xy[illegible]	β [illegible]	D-[illegible]	D-[illegible]	D-[illegible]	D-[illegible]	L-[illegible]	D-[illegible]	L-[illegible]	Gl[illegible]	2-[illegible]	5- Ke
**VR81**	+	+	+	+	-	+	+	-	-	-	-	-	-	+	-	-	-	-	-	-	-	-	-	+
**LVT1**	+	+	+	+	-	+	+	-	-	-	-	-	-	+	-	-	-	-	-	-	-	-	-	+
**R12**	-	-	-	-	+	-	-	-	-	-	-	-	-	-	-	-	-	-	-	-	-	-	-	+
**R15**	-	-	-	-	-	-	-	-	-	-	-	-	-	-	-	-	-	-	-	-	-	-	-	+
**R17**	-	-	-	-	-	-	-	-	-	-	-	-	-	-	-	-	-	-	-	-	-	-	-	+
**OL2**	-	-	-	-	-	-	-	-	-	-	-	-	-	-	-	-	-	-	-	-	-	-	-	+

**ME7**	+	+	+	+	-	+	+	-	-	-	-	-	-	+	-	-	-	-	-	-	-	-	-	+
**ME10**	+	+	+	+	-	+	+	-	-	-	-	-	-	+	-	-	-	-	-	-	-	-	-	+
**ME1**	+	+	+	+	-	+	+	-	-	-	-	-	-	+	-	-	-	-	-	-	-	-	-	+
**ME9**	+	+	+	+	-	+	+	-	-	-	-	-	-	+	-	-	-	-	-	-	-	-	-	+
**ME5**	+	+	+	+	-	+	+	-	-	-	-	-	-	+	-	-	-	-	-	-	-	-	-	+
**ME101**	+	+	+	+	-	+	+	-	-	-	-	-	-	+	-	-	-	-	-	-	-	-	-	+

Table S3. Proteolytic, amylolytic and lipolytic activity of the selected *W. cibaria* and *L. plantarum* strains.

**Strains**	**Proteolytic activity**			**Lipolytic activity**	**Amylolytic activity**
	**2%**	**5%**	**10%**	**2% Tween 80**	**2% Starch**
**R15**	++	++	+++	-	-
**R17**	++	++	+++	-	-
**R12**	++	+++	+++	+	-
**OL2**	+	++	++	-	-
**ME7**	++	++	+++	-	-
**ME10**	+	++	++	-	-
**ME1**	++	++	+++	-	-
**ME9**	++	++	+++	-	-
**ME5**	+	++	++	-	-
**ME101**	+	++	++	-	-
**VR81**	++	+++	+++	-	-
**LVT1**	++	++	+++	-	-

+ ≤ 10 mm; 10 mm < ++ ≤ 20 mm ; +++ > 21

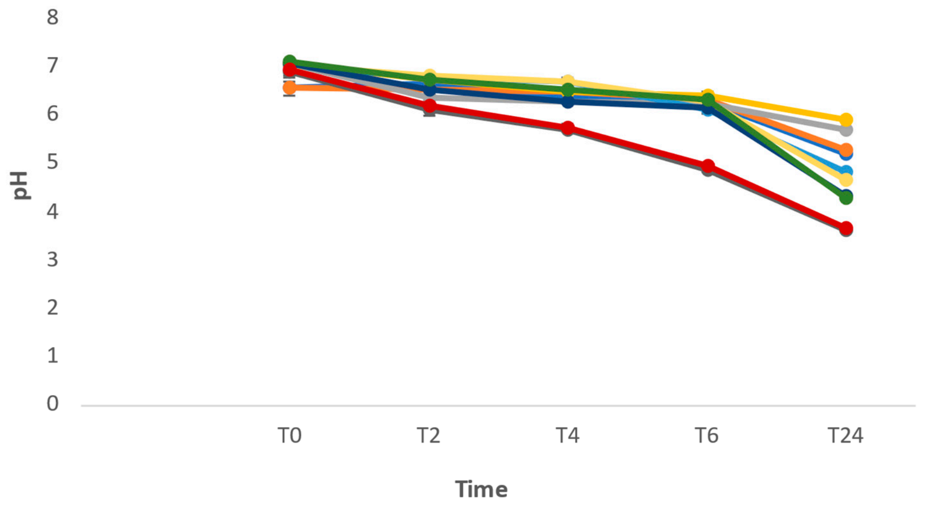

Figure S1. pH variation of skim and plant-based milk using single cultures of *W. cibaria* and *L. plantarum*. Skim milk + *L. plantarum* is represented by the dark blue curve; Skim milk + *W. cibaria* is represented by the orange curve; Cocunut milk + *L. plantarum* is represented by the grey curve; Cocunut milk + *W. cibaria* is represented by the orange curve; Soy milk + *L. plantarum* is represented by the light blue curve; Soy milk + *W. cibaria* is represented by the yellow curve; Oat milk + *L. plantarum* is represented by the black curve; Oat milk + *W. cibaria* is represented by the green curve; Quinoa + *L. plantarum* is represented by the dark grey curve; Quinoa + *W.cibaria* is represented by the red curve. Error bars indicate the standard deviation of triplicate experiments.

Table S4. Exopolysaccharide quantification of the selected LAB strains using sucrose as a sugar source.

LAB strain	Exopolysaccharide mg/ml
**R15**	1,08 ± 0.24
**R17**	-
**R12**	-
**OL2**	-
**ME7**	4,2 ± 0,17
**ME10**	4,12 ± 0.12
**ME1**	4,2 ± 0.28
**ME9**	3,99 ± 0.29
**ME5**	4,54 ± 0.26
**ME101**	4,08 ± 0.06
**VR81**	4 ,7 ± 0.02
**LVT1**	4,6 ± 0.121
